# Involvement of Pore Formation and Osmotic Lysis in the Rapid Killing of Gamma Interferon-Pretreated C166 Endothelial Cells by *Rickettsia prowazekii*

**DOI:** 10.3390/tropicalmed7080163

**Published:** 2022-08-01

**Authors:** Jenifer Turco

**Affiliations:** Biology Department, Valdosta State University, 1500 N. Patterson St., Valdosta, GA 31698, USA; jturco@valdosta.edu

**Keywords:** *Rickettsia prowazekii*, rickettsia, epidemic typhus, interferon, cytokine, endothelial cell, cell death, host response

## Abstract

*Rickettsia prowazekii*, the bacterial cause of epidemic typhus in humans, proliferates mainly within the microvascular endothelial cells. Previous studies have shown that murine macrophage-like RAW264.7 cells are rapidly damaged if they are pretreated with gamma interferon (IFN-γ) and then infected with *R. prowazekii*. In the present study, the effects of IFN-γ and *R. prowazekii* on murine C166 endothelial cells were evaluated. In the IFN-γ-pretreated *R. prowazekii*-infected endothelial cell cultures, evidence of cell damage was observed within several hours after addition of the rickettsiae. Considerable numbers of the cells became permeable to trypan blue dye and ethidium bromide, and substantial amounts of lactate dehydrogenase (LDH) were released from the cells. Such evidence of cellular injury was not observed in the untreated infected cultures or in any of the mock-infected cultures. Polyethylene glycols (PEGs) of different nominal average molecular weights were used to assess the possible involvement of pore formation and osmotic lysis in this cellular injury. PEG 8000 dramatically suppressed LDH release, PEG 4000 partially inhibited it, and PEGs 2000 and 1450 had no effect. Despite its inhibition of LDH release, PEG 8000 did not prevent the staining of the IFN-γ-pretreated infected endothelial cells by ethidium bromide. These findings suggest that the observed cellular injury involves the formation of pores in the endothelial cell membranes, followed by osmotic lysis of the cells.

## 1. Introduction

The genus *Rickettsia* includes several important human pathogens [1,2]. For example, *Rickettsia prowazekii*, the cause of epidemic typhus, and *Rickettsia typhi*, the cause of endemic or murine typhus, are members of the typhus group of the genus. The spotted fever group includes more than 20 species, such as *Rickettsia rickettsii*, the cause of Rocky Mountain spotted fever; *Rickettsia conorii*, the cause of boutonneuse fever (Mediterranean spotted fever); and *Rickettsia parkeri*, the cause of *R. parkeri* rickettsiosis (maculatum disease). Members of a transitional group include *Rickettsia akari*, *Rickettsia australis*, and *Rickettsia felis*, which cause rickettsialpox, Queensland tick typhus, and flea-borne spotted fever, respectively. Rickettsiae are transmitted to humans by arthropod vectors [1,2]. *Rickettsia* species initially enter their host cells through an endocytic process, then quickly exit the endocytic vesicle to reach their intracellular location [1,3,4,5]. The cytoplasm of their host cells is the site of rickettsial growth, and certain rickettsial species may grow within the nucleus as well. A recent article outlined reasons that rickettsial diseases should be acknowledged as “neglected tropical diseases” [6].

Epidemic typhus is a disease associated with human misery and infestation with human body lice, which serve as vectors [1,2,7,8]. This bacterium may be carried by flying squirrels, and has caused disease in humans who had close contact with these animals and/or their lice or fleas. The endothelial cells that line the small blood vessels are important sites of growth of *R. prowazekii* and other *Rickettsia* species in vivo, and damage to these cells occurs, as reviewed by others [9,10,11,12,13,14,15]. However, the mechanisms through which endothelial cell damage occurs are not completely known. *R. prowazekii* and other *Rickettsia* species may grow within macrophages as well, and this ability has been correlated with virulence [16,17,18].

During growth of *R. prowazekii* within the cytoplasm of cultured animal cells such as fibroblasts, large numbers of rickettsiae may fill the cytoplasm before the cells die and release their rickettsiae to infect other cells, as reviewed in [1,4]. This rickettsial proliferation generally occurs over a period of days. Virulent strains of *R. prowazekii* similarly accumulate during a period of days in cultured human macrophages [16] and mouse macrophage-like cells [17,19]. However, gamma interferon (IFN-γ) makes murine macrophage-like RAW264.7 cells exquisitely sensitive to being rapidly killed by much smaller numbers of viable *R. prowazekii* bacteria [19,20,21]. When these cells are pretreated with IFN-γ and then infected (with either virulent or avirulent *R. prowazekii*), many of the macrophages die (as shown by their uptake of trypan blue dye) within several hours after infection. Cultures of mouse L929 cells show evidence of premature cellular injury after being exposed to the combination of IFN-γ treatment plus *R. prowazekii* infection; however, the damage appears more slowly than in macrophage-like cells [22]. The responses of cultured murine endothelial cells to the combination of IFN-γ treatment and *R. prowazekii* infection have not been determined.

Despite these observations of host cell injury, IFN-γ treatment of cultured cells is associated with inactivation of *R. prowazekii* and suppression of its intracellular growth [19,20,21,22,23,24]. Furthermore, animal studies with different rickettsial species have demonstrated the importance of IFN-γ as an antirickettsial host defense [25,26,27]. Nevertheless, the ability of IFN-γ to sensitize cultured host cells to early injury after *R. prowazekii* infection raises the possibility that this cytokine might contribute to cellular damage in an infected host [19,20,21,22].

Due to their importance in normal development and roles in various diseases, cell death and the mechanisms involved in its various forms (such as apoptosis, pyroptosis, necrosis, necroptosis, and others) have been extensively studied [28,29,30]. Several types of regulated (programmed) cell death, as well as autophagy (which may sometimes be associated with cell death), are among the innate host defenses against pathogens, and intracellular pathogens may manipulate the processes involved in cell death in ways that aid their own survival and replication, as reviewed in [31,32,33,34,35,36]. For example, activation of the transcription factor NF-κB inhibits endothelial cell death due to apoptosis in R. rickettsii–infected cultures [37,38]. In addition, R. parkeri activates the inflammasome and induces pyroptosis in bone marrow-derived macrophages, thus avoiding the effects of type I interferon [39]. Activation of inflammasome-dependent release of protective interleukin-1α (IL-1α) by mouse macrophages appears to be inversely correlated with pathogenicity, as R. montanensis (a non-pathogenic rickettsia) triggered the release of IL-1α, while pathogenic rickettsiae (R. typhi and R. rickettsii) did not [40]. Other examples involve the findings that avoidance of autophagy by R. parkeri is associated with its outer membrane protein B (OmpB) protein [41] and with methylation of lysine residues on this protein [42]. In a final example, researchers showed that infection of HMEC-1 endothelial cells with a highly pathogenic strain of R. conorii was associated with substantial caspase-1-dependent host cell damage after three days, whereas such damage was not observed when the cells were infected with R. massiliae, which causes only a mild spotted fever [43].

Formation of pores or channels in the cytoplasmic membrane contributes to the injury of human and animal cells affected by certain forms of cell death (such as pyroptosis and necroptosis) [44,45,46] or exposed to toxins (or other proteins) that form pores in membranes [47,48,49]. Certain pore-forming toxins can actually trigger pyroptosis or necroptosis [48].

Polyethylene glycols (PEGs) of different molecular weights have been used to evaluate the roles of pore formation and osmotic lysis in cell death [50,51,52,53,54]. PEGs protect cells from osmotic lysis if they are sufficiently large to be excluded from cells that were initially damaged by pore formation [50,51]. This approach allows estimation of the diameter of the membrane pores based on the hydrodynamic radii of the PEGs.

Because endothelial cells are important sites of *R. prowazekii* growth in vivo, this study first sought to determine whether IFN-γ-pretreated cultured endothelial cells would be rapidly killed by *R. prowazekii.* The results indicated that IFN-γ-pretreated endothelial cells were killed within hours after infection; they became permeable to trypan blue dye and to ethidium bromide, and released the intracytoplasmic enzyme lactate dehydrogenase (LDH). Subsequent experiments used PEGs of different molecular weights as osmoprotectants to evaluate the possible roles of pore formation and osmotic lysis in the killing of the endothelial cells. Although PEG 8000 was very effective at preventing the cells from releasing LDH, it did not eliminate their uptake of ethidium bromide, a small molecule that may be used to assess the presence of membrane pores. These findings indicate that IFN-γ-pretreated, cultured endothelial cells are rapidly killed after *R. prowazekii* infection, supporting the hypothesis that pore formation and osmotic lysis are involved in this cellular injury. Moreover, this study contributes to an understanding of how the combination of IFN-γ pretreatment and rickettsial infection leads to early host cell death, and may facilitate additional research to further delineate the responsible mechanisms.

## 2. Materials and Methods

### 2.1. Cultured Cells, IFN-γ, PEGs, Rickettsiae, and Control L929 Cell Preparation

#### 2.1.1. Cultured Cells and IFN-γ

The mouse endothelial C166 cell line (ATCC CRL 2581) was purchased from the American Type Culture Collection (ATCC, Manassas, VA, USA) and grown in high glucose Dulbecco’s Modified Eagle medium supplemented with nonessential amino acids and sodium pyruvate (DULNeaaNaP) plus 10% fetal bovine serum (FBS). The mouse fibroblastic L929 cell line was provided by Jonathon Audia and Herbert Winkler (University of South Alabama, Mobile, AL, USA). This cell line was originally purchased from Flow Laboratories [55]. L929 cells were grown in Eagle Minimal Essential Medium (MEM) plus 10% newborn bovine serum. All cell cultures were maintained at 34.5 °C in a humidified incubator with 5% carbon dioxide in air. Bovine sera were purchased from Thermo Fisher Scientific, Waltham, MA, USA, or Cytiva Life Sciences, Marlborough, MA, USA. Murine recombinant IFN-γ (4.7 × 10^6^ units/mg) was provided by Genentech, Inc, South San Francisco, CA, USA.

#### 2.1.2. PEGs

Polyethylene glycols with different nominal average molecular weights (PEGs 8000, 4000, 2000, and 1450) were purchased from Fisher Scientific, Pittsburgh, PA, USA, or Sigma-Aldrich, Inc., St. Louis, MO, USA. For use in experiments, PEGs were dissolved in phenol red-free DULNeaaNaP plus 5% FBS (hereafter designated FDS5%) and filter-sterilized by being passed through filters with a pore size of 0.2 µm. PEGs 4000, 2000, and 1450 were used at a concentration of 30 mM, whereas PEG 8000 was used at a concentration of 15 mM due to its more limited solubility. Fresh PEG solutions were prepared for each experiment.

#### 2.1.3. Rickettsiae and Control L929 Cell Preparation

Initial samples of the Madrid E strain of *R. prowazekii* were generously provided by Jonathon Audia and Herbert Winkler (University of South Alabama, Mobile, AL, USA). All work with *R. prowazekii* was conducted in a certified Biosafety Level 3 facility in accordance with regulations of the United States Government Select Agent Program.

Rickettsial preparations for experimental use were generated from infected L929 cells essentially as previously described [56]. Confluent L929 cells from 4–250 cm^2^ tissue culture flasks were harvested, centrifuged, and resuspended in Hank’s balanced salt solution supplemented with 0.1% gelatin and 5 mM potassium glutamate. After addition of *R. prowazekii* Madrid E, the tube was centrifuged at 1000× *g* for 15 min at room temperature to facilitate infection. The tube was then mixed briefly on a vortex mixer and incubated at 37 °C for at least one hour, with periodic hand mixing. After centrifugation of the tube at 1000× *g* for 7 min at room temperature, the pellet was resuspended in MEM plus 10% newborn bovine serum, and the cells were distributed into 4–150 mm diameter tissue culture plates. Samples of the cells were dispensed into the wells of a six-well plate that contained coverslips, allowing the rickettsial infection to be checked periodically. The infected cultures were incubated at 34.5 °C in a humidified incubator with 5% carbon dioxide in air. The following day, cycloheximide (1 µg/mL) was added to inhibit host cell protein synthesis and growth. When the L929 cells were heavily infected (generally after incubation for about two additional days), the rickettsiae were harvested as follows. The culture media were collected in sterile tubes and the adherent L929 cells were washed once with a sucrose–phosphate glutamate solution (SPG) (0.218 M sucrose, 3.76 mM KH_2_PO_4_, 7.1 mM K_2_HPO_4_, 4.9 mM potassium glutamate, pH 7.0), then the washes were combined with the culture media. The culture media plus washes were then centrifuged at 9000× *g* for 10 min at 5 °C to collect any detached cells and released rickettsiae. After removal of the supernatant fluid, each pellet was resuspended with SPG and the mixtures were combined in a single tube (A) that was placed on ice. The adherent washed L929 cells in the plates were scraped into 3 mL of SPG per dish and then transferred to the same tube (A). The plates were then washed and the washes were added to the tube. Next, the tube was centrifuged at 9000× *g* for 10 min at 5 °C and the pellet was suspended in 3 mL SPG. Three milliliters of sterile glass beads (1 mm diameter) were added and the mixture was vortexed to rupture the cells. The suspension was kept cool during vortexing by alternating periods of vortexing with periods of cooling on ice. The total vortexing time was 4 min. The beads were allowed to settle, and the homogenate was transferred to a separate tube. Next, the beads were washed with SPG and the washes were added to the homogenate. The combined homogenate and washes were centrifuged at 300× *g* for 5 min, then the supernatant fluid was removed to another tube and centrifuged at 9000× *g* for 10 min at 5 °C. Finally, the resultant pellet was resuspended in about 10 mL SPG and dispensed into vials that were frozen in liquid nitrogen. The vials were stored in a liquid nitrogen storage container (vapor phase) or in a freezer at −80 °C. For use as a control, uninfected L929 cells were processed and frozen in a similar manner.

Dilutions of rickettsial preparations used in these experiments ranged from 1/200 to 1/1000. In certain instances, a control preparation made from uninfected L929 cells was tested at a dilution of 1/10 or 1/20.

### 2.2. Preparation of Cultures of Untreated and IFN-γ-Pretreated C166 Endothelial Cells, Mock Infection or Infection of the C166 Cells with Rickettsiae, Evaluation of the Infection, and Assessment of Damage to the Endothelial Cells in the Absence or Presence of PEGs

#### 2.2.1. Preparation of Untreated and IFN-γ-Pretreated C166 Endothelial Cells

Circular glass coverslips (12 mm diameter; #1) were placed in some wells of 24-well tissue culture plates. Monolayers of C166 cells were treated with a mixture of 0.25% trypsin and 0.01% ethylenediaminetetraacetic acid (disodium salt) in phosphate-buffered saline (pH 7.6) to release the cells, which were suspended in approximately 20 mL DULNeaaNaP plus 10% FBS and then centrifuged at 200× *g* for 7 min at room temperature. After removal of the supernatant fluid, the cells were resuspended in DULNeaaNaP plus 5% FBS, adjusted to a concentration of 1.6 × 10^5^ viable (trypan blue-excluding) cells per ml, and dispensed in the wells of the 24-well plates (0.4 mL per well). The cells were incubated overnight; then, 0.1 mL of recombinant IFN-γ diluted in DULNeaaNaP plus 5% FBS was added to some wells so the final concentration of IFN-γ would be 25 units per ml. Untreated wells were given 0.1 mL of DULNeaaNaP plus 5% FBS.

#### 2.2.2. Mock Infection or Infection of the C166 Cells with Rickettsiae in the Absence or Presence of PEGs

After being incubated for 22 to 25 h, the cells were washed once with phenol red-free DULNeaaNaP plus 5% FBS (hereafter designated FDS5%). For mock-infection or infection, respectively, FDS5% alone or rickettsiae diluted in FDS5% (0.5 mL per well) was added. To facilitate the infection process, the plates were centrifuged at 500× *g* for 15 min at room temperature, then incubated at 34.5 °C for 45 min. The coverslips were removed and air-dried to allow assessment of the rickettsial infection. In certain experiments, PEGs were added during the rickettsial infection at the previously stated concentrations. In other instances, the medium was removed and medium with or without PEGs was added either after the 15-min centrifugation period or after subsequent incubation for 45 min (1 h after addition of the rickettsiae).

#### 2.2.3. Evaluation of Rickettsial Infection

Rickettsial infection was evaluated in experiments after staining the cultured cells, essentially as described previously in [55,57]. Briefly, the C166 cells on the air-dried coverslips were fixed for 20 min at room temperature in a solution of 0.37% formaldehyde in 0.1 M sodium phosphate buffer, pH 6.8. After being washed in the same buffer four times, the cells were then stained according to the method of Giménez [58]. The stained cells were examined with the oil immersion objective of a light microscope and the numbers of rickettsiae present in each of 100 C166 cells on each coverslip were determined. The percentage of cells infected, rickettsiae per infected cell, and rickettsiae per cell were calculated. This method of staining was used to empirically determine the dilutions of rickettsiae for use in experiments. 

#### 2.2.4. Assessment of Damage to the Endothelial Cells with Trypan Blue Dye

For evaluation of endothelial cell viability, in certain experiments the medium was removed from each well and replaced with 0.08% trypan blue diluted in fresh DULNeaaNaP plus 5% FBS. After incubation of the cells for 5 to 10 min at 34.5 °C, the dye solution was removed and replaced with sterile warm phosphate-buffered saline. The cells were then examined with an inverted bright field microscope; at least 100 cells were scored as viable (unstained) or dead (stained) in each well. Trypan blue staining was generally performed at 4 h after addition of the rickettsiae.

#### 2.2.5. Evaluation of Damage to the Endothelial Cells in the Absence or Presence of PEGs at Various Times after Mock-Infection or Infection

Two methods were used to evaluate endothelial cell damage in the absence or presence of PEGs: staining of the endothelial cells with ethidium bromide (which does not stain healthy cells) and assay of the cell culture media for the cytoplasmic enzyme, LDH, which is released by heavily damaged cells. In these experiments, FDS 5% was used as described in Section 2.2.2. Staining with ethidium bromide (Fisher Scientific) was generally performed 3 to 6 h after addition of the rickettsiae. Culture media were removed at various times for LDH assays; however, in most experiments the media were removed at 3 or 4 h after addition of the rickettsiae.

For staining, ethidium bromide diluted in FDS 5% was added to each well to make up a final concentration of 25 µg/mL. The contents of each well were then mixed gently with a pipette. After incubation for 10 min at 34.5 °C, the cells were examined in an EVOS FL inverted microscope (Thermo Fisher Scientific). For each well, separate images were recorded for transmitted light and fluorescence; in certain experiments, an overlay of these two images was recorded as well. Transmitted light and overlay images shown in this study were adjusted for exposure and contrast using Adobe Photoshop CC 2018^®^ software (Adobe Inc., San Jose, CA, USA). In all cases, the same adjustments were applied to an entire image. The fluorescence images shown in this study were not adjusted. However, Figures S1 and S2 show both the unadjusted fluorescence images and the corresponding fluorescence images that were adjusted for exposure and contrast using the same settings applied to the transmitted light and overlay images.

For LDH assays, Pierce LDH Cytotoxicity Assay Kits were purchased from Thermo Fisher Scientific. The assay was performed in flat-bottomed 96-well plates, essentially as described by the manufacturer, with the following modification. After the assay was completed, 20 µL of 37% formaldehyde was added to each well (final formaldehyde concentration, 4.3%), then the plate was covered, mixed, and incubated for 10 min at room temperature to allow inactivation of the rickettsiae. The results of the assay were then read on a SpectraMax M5e Microplate Reader (MDS Analytical Technologies, Sunnyvale, CA, USA).

### 2.3. Statistical Analyses

The Assistant in Minitab^®^ 20 Statistical Software (Minitab, State College, PA, USA) was used to perform a one-way analysis of variance for the various datasets.

## 3. Results

### 3.1. Substantial Percentages of IFN-γ-Pretreated C166 Endothelial Cells Were Damaged within Four Hours after Addition of R. prowazekii

#### 3.1.1. Results of Assessment of Endothelial Cell Damage by Trypan Blue Staining

**Figure 1:**
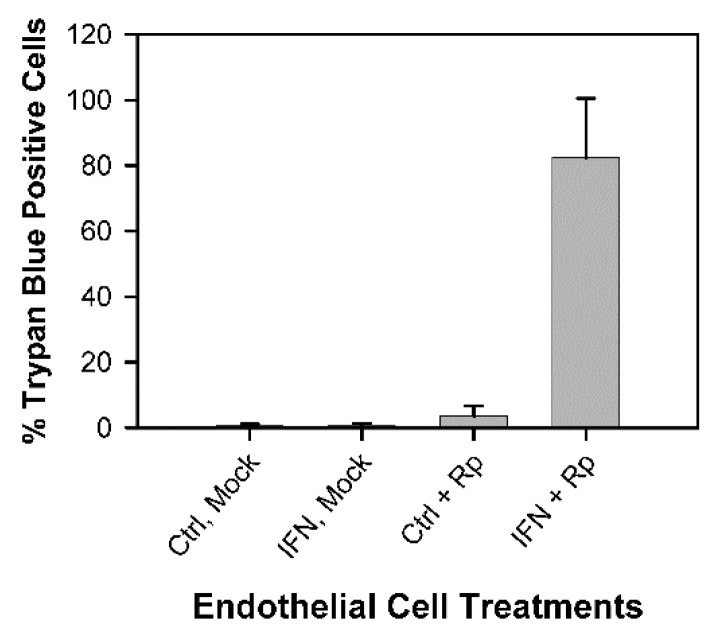
IFN-γ-pretreated cultures of C166 endothelial cells showed increased permeability to trypan blue dye within 4 h after addition of *R. prowazekii*. Data were collected in twelve independent experiments. Each bar represents the mean ± standard deviation (SD) for the following numbers of determinations: untreated mock-infected cultures (Ctrl, Mock), n = 19; IFN-γ-pretreated mock-infected cultures (IFN, Mock), n = 21; untreated *R. prowazekii*-infected cultures (Ctrl + Rp), n = 24; IFN-γ-pretreated *R. prowazekii*-infected cultures (IFN + Rp), n = 26. The IFN-γ-pretreated infected cultures differed significantly from the other cultures (*p* < 0.01). At 1 h after addition of the rickettsiae, the percentages of cells infected were 94.9 ± 6.1 and 96.4 ± 6.3, and the numbers of rickettsiae per infected cell were 13.6 ± 4.8 and 15.9 ± 6.4 in the untreated and IFN-γ-pretreated cultures, respectively (mean ± SD, n = 16) (Table S2).

Many of the IFN-γ-pretreated infected endothelial cells became permeable to trypan blue dye (Figure 1 and Table S1), which did not stain substantial numbers of cells in the untreated infected cultures, the untreated mock-infected cultures, or the IFN-γ-pretreated mock-infected cultures. This endothelial cell damage was observed within 4 h after addition of the rickettsiae to the endothelial cells. Thus, IFN-γ-pretreated C166 endothelial cells, like IFN-γ-pretreated macrophage-like RAW265.7 cells [19,20,21,23], were rapidly damaged after infection with *R. prowazekii*. The dilutions of the rickettsial preparations used in the experiments with trypan blue ranged from 1/200 to 1/1000. As a control, a lysate of uninfected L929 cells that had been processed in the same manner as the rickettsial preparations was used at a dilution of 1/10 or 1/20. The percentages of trypan blue positive cells (means ± standard deviations) were 9.4 ± 2.0 (1/10, n = 3) and 3.6 ± 0.2 (1/20, n = 2) for the untreated cultures and 10.9 ± 2.4 (1/10, n = 3) and 3.4 ± 0.6 (1/20, n = 2) for the IFN-γ-pretreated cultures (Table S1). These data indicate that the rickettsiae, rather than other component(s) in the L929 cell lysates, were responsible for the majority of the damage caused by the rickettsial preparations.

#### 3.1.2. Results of Assessment of Endothelial Cell Damage by Ethidium Bromide Staining

**Figure 2:**
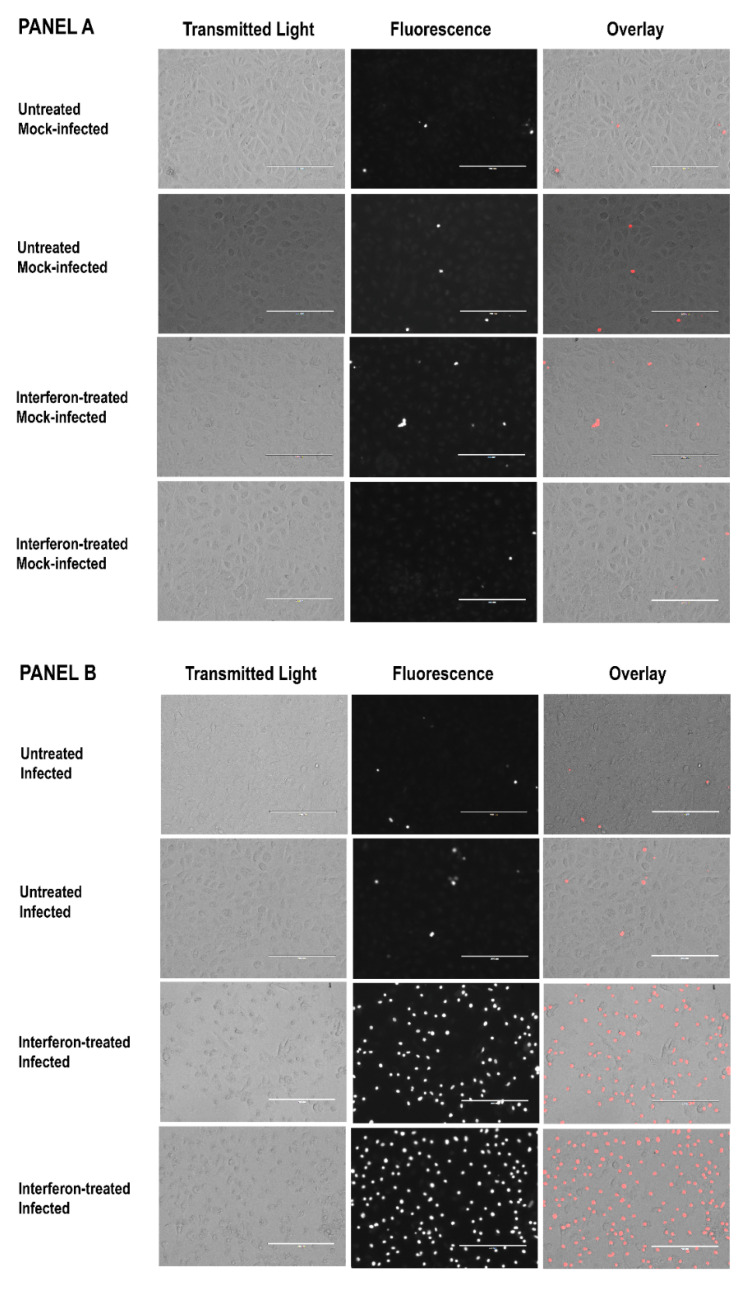
Results of ethidium bromide staining of untreated and IFN-γ-pretreated endothelial cells 3 to 6 h after the start of the mock infection (Panel **A**) or after addition of *R. prowazekii* (Panel **B**). The micrographs show duplicate cultures from a representative experiment. Figure S1 shows all images from the experiment, and indicates which images are shown here. Each bar represents 200 µm. To eliminate concern about image manipulation, all transmitted light images were captured under identical conditions, and all fluorescence images were captured under identical conditions as well. All transmitted light micrographs and all overlays were then adjusted for exposure and contrast using identical settings in Adobe Photoshop CC 2018^®^ software.

The results of the experiments that used ethidium bromide to assess endothelial cell damage had a similar pattern (Figure 2 and Figure S1). Cultures of IFN-γ-pretreated C166 endothelial cells showed increased permeability to ethidium bromide within 3 to 6 h after addition of *R. prowazekii* (Figure 2, Panel B, bottom two rows). Few cells were stained by ethidium bromide in the mock-infected cultures (Figure 2, Panel A) or in the untreated infected cultures (Figure 2, Panel B, top two rows).

**Figure 3:**
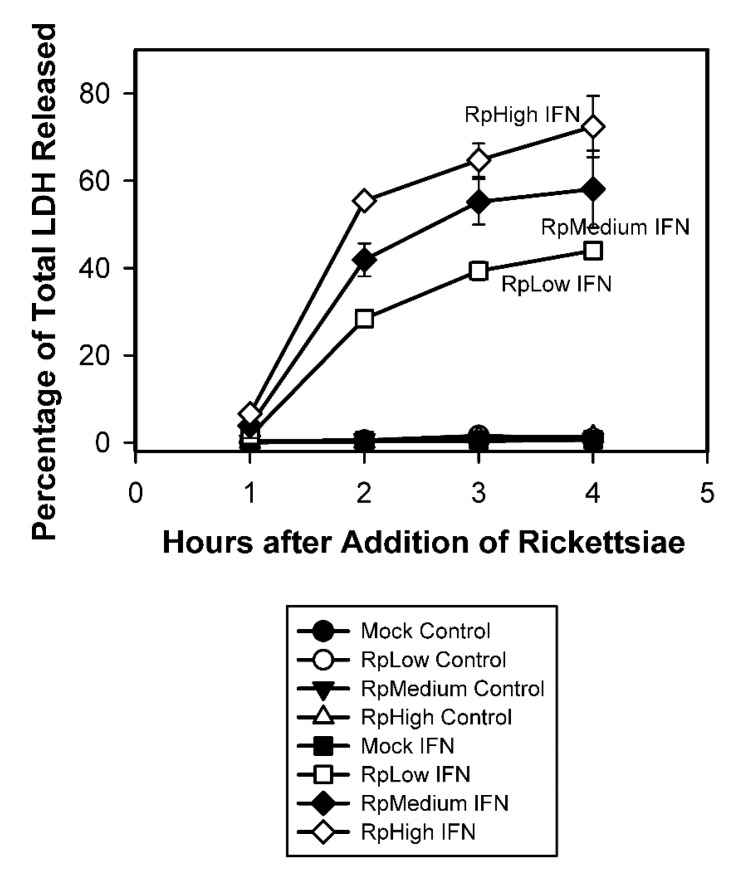
Release of lactate dehydrogenase (LDH) by IFN-γ-pretreated C166 endothelial cells after addition of *R. prowazekii* (Rp). Each value represents the mean ± standard deviation; the number of determinations for each point is shown in Table S3. Individual data points are shown in Table S4. At one hour after addition of the rickettsiae, the rickettsial infections were determined by microscopic counting of stained cells from triplicate cultures for each dilution of the rickettsial preparation used (1/900, RpLow; 1/450, RpMedium; and 1/200, RpHigh). The percentages of cells infected (means ± standard deviations) were 64.0 ± 9.5, 84.3 ± 5.9, and 94.3 ± 5.5, respectively, in the untreated cultures and 78.0 ± 4.4, 93.3 ± 3.2, and 96.0 ± 1.0, respectively, in the IFN-γ-pretreated cultures. The numbers of rickettsiae per infected cell were 3.7 ± 0.7, 6.9 ± 0.9, and 13.7 ± 3.7, respectively, in the untreated cultures and 6.0 ± 1.3, 9.2 ± 2.5, and 15.1 ± 1.5, respectively, in the IFN-γ-pretreated cultures (Table S5). In the panel below the figure, “Control” indicates the untreated cultures and “IFN” indicates the IFN-γ-pretreated cultures. In certain instances, the values for the standard deviations were within the symbols.

In addition to exhibiting permeability to trypan blue and ethidium bromide (which are small molecules of 961 and 394 daltons, respectively), the IFN-γ-pretreated infected endothelial cells released substantial amounts of the large cytoplasmic enzyme lactate dehydrogenase (approximately 140 kilodaltons) into the culture medium (Figure 3 and Table S3). At the 4 h time point, the mean values for the IFN-γ-pretreated cultures infected with different concentrations of rickettsiae differed from one another and from the mean for the IFN-γ-pretreated mock-infected cultures (*p* < 0.05). Substantial release of LDH did not occur in the IFN-γ-pretreated mock-infected cultures, the untreated mock-infected cultures, or the untreated infected cultures (Figure 3 and Table S3).

### 3.2. Effect of Polyethylene Glycols (PEGs) with Various Nominal Average Molecular Weights on the Release of LDH by IFN-γ-Pretreated C166 Endothelial Cells after Infection with R. prowazekii

**Figure 4:**
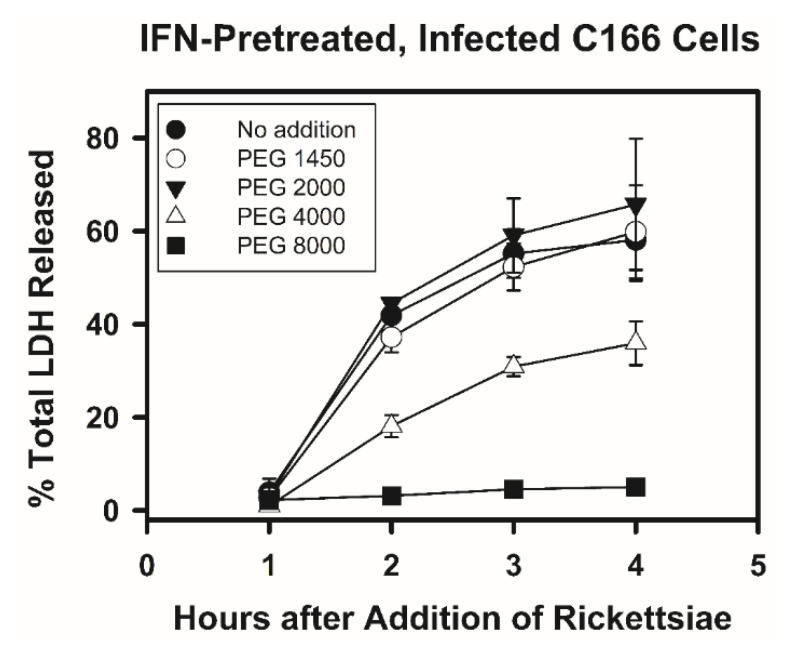
Effect of PEGs of different nominal average molecular weights on the release of lactate dehydrogenase (LDH) from IFN-γ-pretreated C166 endothelial cells after addition of *R. prowazekii*. PEGs were added to IFN-γ-pretreated and washed cells along with rickettsiae and samples of the culture media were collected for assay of LDH activity at various times. Table 1 provides the number of determinations and shows additional data from these experiments. Table S4 provides the individual data points. For PEG 8000, the values for the standard deviations were within the symbols. PEGs were used at a concentration of 30 mM, except for PEG 8000, which was used at a concentration of 15 mM. At 2 h, 3 h, and 4 h after addition of the rickettsiae, the mean value for the cultures without PEG differed significantly from the mean values for the cultures with PEG 8000 or PEG 4000 (*p* < 0.05).

In order to determine whether pore formation and osmotic lysis contributed to the death of the IFN-γ-pretreated *R. prowazekii*-infected endothelial cells, the ability of different PEGs to protect the IFN-γ-pretreated infected endothelial cells was evaluated (Figure 4, Table 1 and Table S4). As noted previously, PEGs can prevent osmotic lysis and the associated release of LDH if they are large enough to be retained outside of the initially damaged cells [50,51]. The data revealed that PEG with an average nominal molecular weight of 8000 (PEG 8000, 15 mM) dramatically suppressed the release of LDH from IFN-γ-pretreated infected endothelial cells, whereas PEG 4000 (30 mM) only partially inhibited LDH release. In contrast, smaller PEGs (PEGs 2000 and 1450, 30 mM) did not inhibit LDH release. Figure 4 shows the data for only the IFN-γ-pretreated infected cultures, whereas Table 1 and Table S4 are more inclusive and show the additional data for the IFN-γ-pretreated mock-infected cultures, the untreated mock-infected cultures, and the untreated infected cultures both with and without PEGs. None of these released substantial amounts of LDH.

As previously noted, PEG 4000 was generally used at a concentration of 30 mM, whereas PEG 8000 was used at 15 mM due to its more limited solubility. In a few instances, lower concentrations of these PEGs were tested (Table S6); the PEGs were added along with the rickettsiae and were continuously present thereafter. For PEG 8000, a concentration of 7.5 mM failed to prevent the release of LDH from IFN-γ-pretreated *R. prowazekii*-infected endothelial cells. Similarly, for PEG 4000, concentrations of 15 mM and 7.5 mM failed to suppress the release of LDH from IFN-γ-pretreated *R. prowazekii*-infected endothelial cells (Table S6). Additional data for untreated cultures may be viewed in Table S4. For statistical analysis, the data for each treatment at the 3 h and 4 h time points were combined. The only IFN-γ-pretreated infected cultures that showed a significant reduction in the mean values for the percentage of total LDH released compared with the IFN-γ-pretreated infected cultures without PEG were those with PEG 4000 (30 mM) or PEG 8000 (15 mM) (*p* < 0.05). The effectiveness of PEG 8000 at suppressing the release of LDH was observed with products from two different suppliers.

To determine whether PEG 8000 might be affecting the interactions between the rickettsiae and the IFN-γ-pretreated host cells, the time of addition of the PEG 8000 was varied (Table 1 and Table S4). For example, in certain instances PEG 8000 was added 15 min after the rickettsiae (after the 15-min period of centrifugation), while in other cases PEG 8000 was added after incubating the cells for an additional 45 min (1 h after addition of the rickettsiae). The data show that PEG 8000 was very effective at preventing the release of LDH from the IFN-γ-pretreated infected endothelial cells regardless of the time of addition (Table 1 and Table S4). Likewise, PEG 4000 partially suppressed LDH release from the IFN-γ-pretreated infected cells irrespective of whether it was added along with the rickettsiae or 1 h later. These data suggest that PEGs 8000 and 4000 are unlikely to exert their effects on LDH release through interference with the initial rickettsial infection process. An effort was made to use staining of the rickettsiae to evaluate the level of the rickettsial infection when the endothelial cells were infected in the presence of PEG 8000 or PEG 4000. Unfortunately, however, these PEGs interfered with the rickettsial staining procedure used in this study. Therefore, adding the PEGs up to 1 h after the addition of the rickettsiae was the approach used to address this question.

In other cases, PEG 8000 was removed from certain IFN-γ-pretreated infected cultures at 3 to 4 h after addition of the rickettsiae, then the cultures were washed and incubated for an additional 3 to 3.5 h in medium without PEG 8000 (Table S7). In these cultures, the mean percentage of total LDH released was 21.8% ± 1.5% (n = 5), whereas the percentage of total LDH released was significantly lower (2.5% ± 0.1%, *p* < 0.05, n = 2) in similar cultures (IFN-γ-pretreated infected cultures with PEG 8000) that were washed and given fresh medium containing PEG 8000. Although the mean percentage of LDH released in the former cultures was significantly lower than the corresponding means for the IFN-γ-pretreated infected cultures at 3 and 4 h after addition of the rickettsiae (55.2% ± 5.2% and 58.1% ± 8.8%, respectively, *p* < 0.05; Figure 4 and Table 1), it was significantly higher than the mean for the cultures maintained continuously in the presence of PEG 8000 (2.5% ± 0.1%, *p* < 0.05). These data suggest that the PEG 8000 protects the endothelial cells rather than having some nonspecific inhibitory effect on the rickettsia–host cell interaction. It is important to note that the IFN-γ-pretreated infected endothelial cells from which the PEG 8000 was removed had been without IFN-γ for about 7 h when the media were collected for assay of LDH.

### 3.3. Inability of PEG 8000 to Prevent the Uptake of Ethidium Bromide by IFN-γ-Pretreated, R. prowazekii-Infected Endothelial Cells

**Figure 5:**
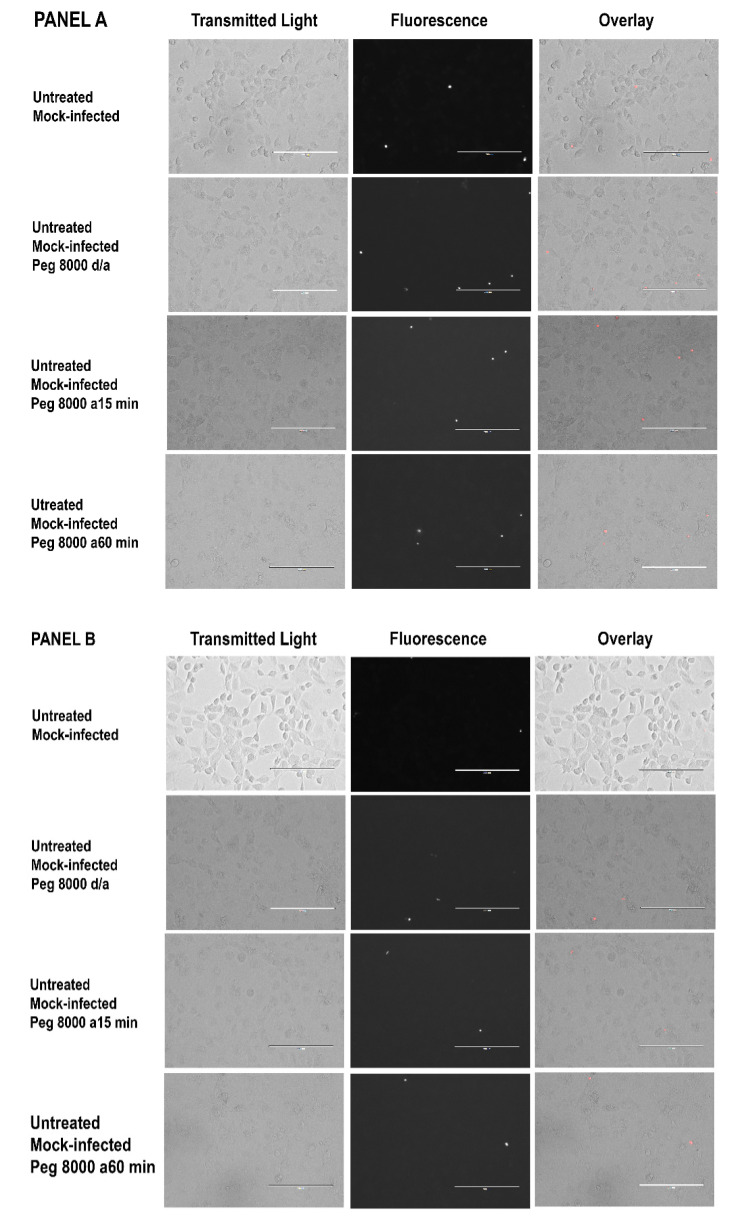

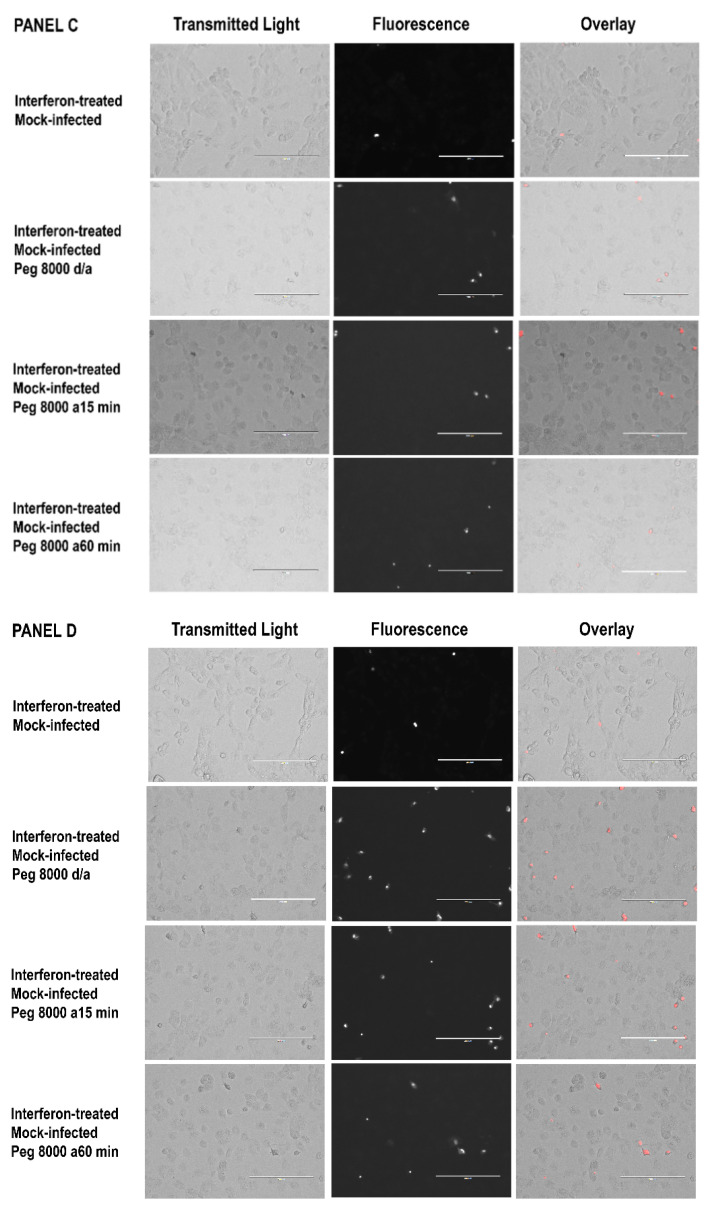

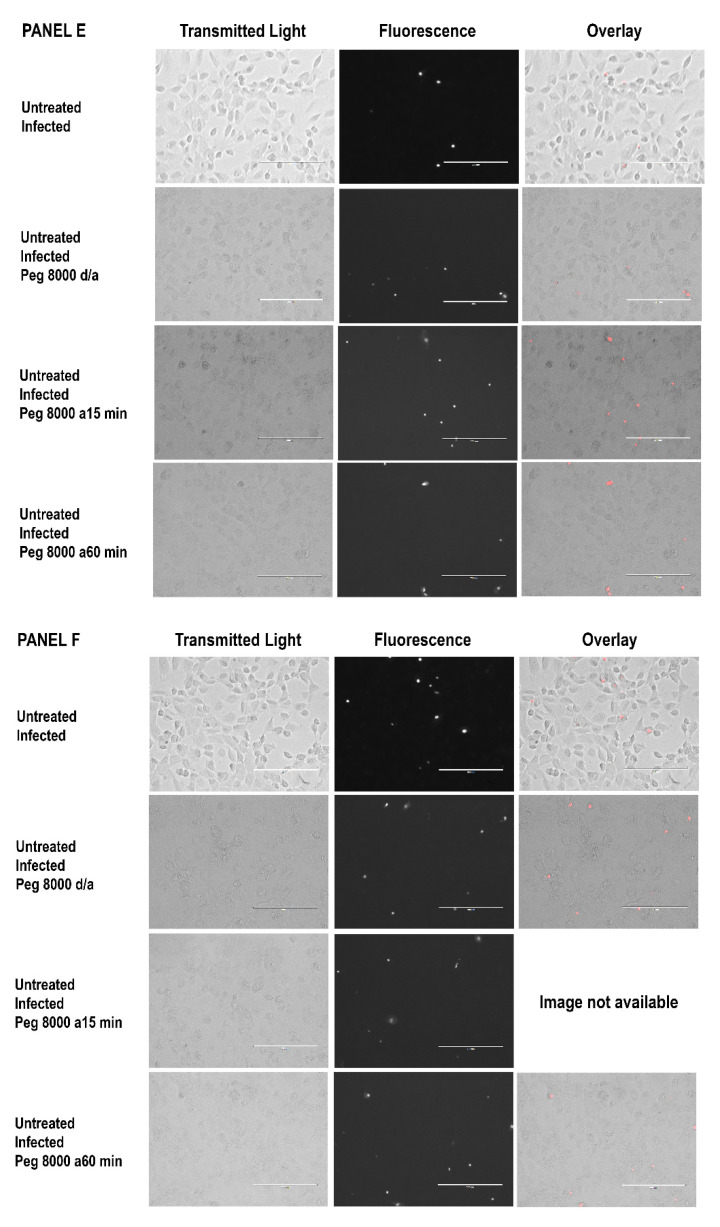

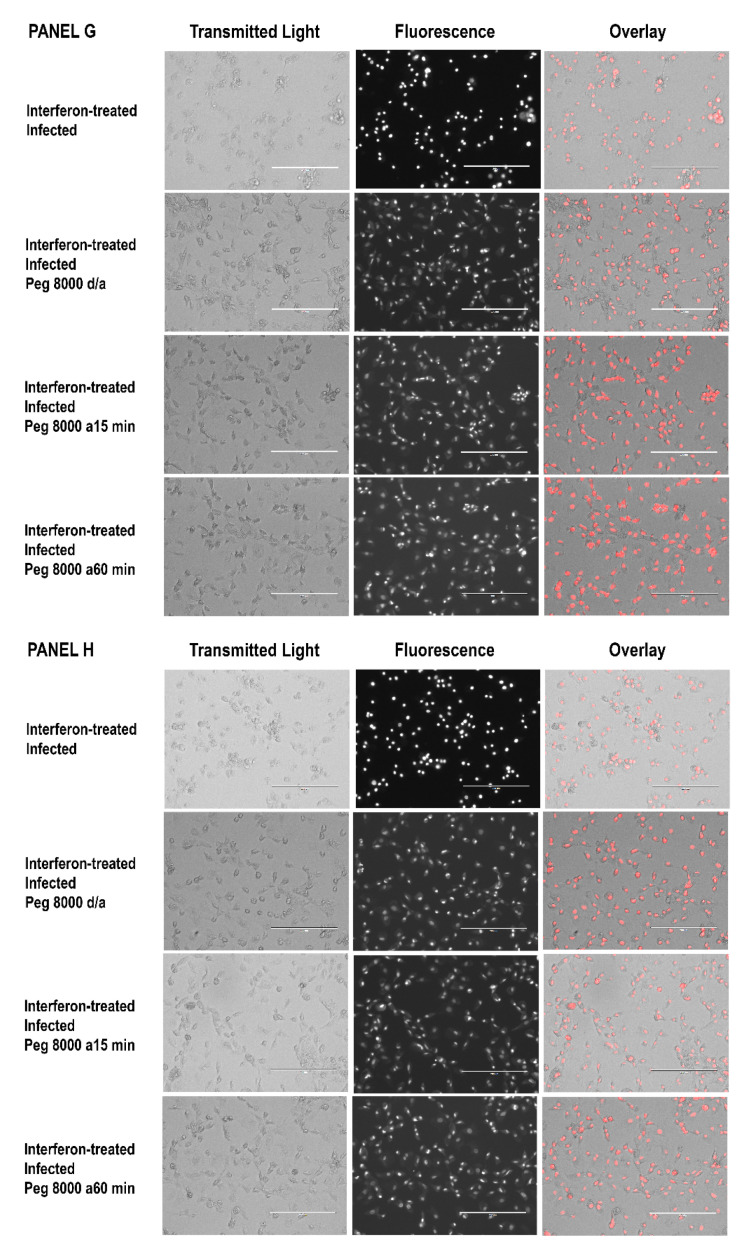
Results of ethidium bromide staining of untreated and IFN-γ-pretreated endothelial cells 3 to 6 h after the start of the mock infection (Panels **A**–**D**) or after addition of *R. prowazekii* (Panels **E**–**H**). d/a, PEG 8000 (15 mM) was added during the mock infection or rickettsial infection and was continuously present thereafter; a15 min, PEG 8000 was added after the 15-min centrifugation; a60 min, PEG 8000 was added 60 min after the start of the mock infection or rickettsial infection. The micrographs in all panels show duplicate cultures from a representative experiment. Figure S2 shows all images from the experiment and indicates which images are shown here. Each bar represents 200 µm. To eliminate concern about image manipulation, all transmitted light images were captured under identical conditions, and all fluorescence images were captured under identical conditions as well. All transmitted light micrographs and all overlays were then adjusted for exposure and contrast using identical settings in Adobe Photoshop CC 2018^®^ software.

Despite the ability of PEG 8000 to suppress the release of LDH from the IFN-γ-pretreated *R. prowazekii*-infected C166 endothelial cells, PEG 8000 did not prevent the cells from being stained by ethidium bromide, irrespective of whether it was added at the same time as the rickettsiae or later (Figure 5, panels G and H; Figure S2). In contrast, ethidium bromide did not stain substantial numbers of cells in the untreated mock-infected cultures (Figure 5, panels A and B; Figure S2), the IFN-γ-pretreated mock-infected cultures (Figure 5, panels C and D; Figure S2), or the untreated *R. prowazekii*-infected cultures (Figure 5, panels E and F; Figure S2), regardless of when the PEG 8000 was added. Figure S1 shows comparable results for a similar independent experiment, and Figure S3 shows images from an additional independent experiment. The images in Figure S3 have a better visual appearance because adjustments were made to optimize the appearance of each image using Adobe Photoshop CC 2018^®^ software.

The ethidium bromide staining of the IFN-γ-pretreated, *R. prowazekii*-infected, and PEG 8000-treated endothelial cells suggested that pores were present in the cytoplasmic membranes of these cells. In order to estimate the diameters of the pores, the hydrodynamic radii of the PEGs used in these experiments were calculated as described by other researchers [59]. The hydrodynamic radii were PEG 1450, 1.2 nm; PEG 2000, 1.41 nm; PEG 4000, 2.0 nm; and PEG 8000, 2.83 nm; thus, the corresponding diameters were, respectively, 2.4 nm, 2.82 nm, 4.0 nm, and 5.66 nm. In order for a PEG to protect cells from osmotic lysis, its size must be greater than the size of the pores in the membrane, as the PEG will be excluded from entering the damaged cells [50,51]. Because PEG 2000 (diameter of 2.82 nm) did not suppress the release of LDH, it follows that the PEG 2000 must have entered the cells, which likely had pores that were larger than 2.82 nm in diameter. Similarly, because PEG 8000 (diameter of 5.66 nm) was highly effective at preventing the release of LDH, it must have been excluded from the damaged endothelial cells, which likely had pores with diameters smaller than 5.66 nm.

## 4. Discussion

Previous studies have documented the synergistic cytotoxic effect of the combination of IFN-γ treatment and *R. prowazekii* infection on cultured mouse macrophage-like RAW264.7 cells and cultured mouse L929 cells [19,20,21,22,23]. Cell damage occurs within hours after *R. prowazekii* infection in cultures of IFN-γ-pretreated RAW264.7 cells, but is slower to appear in infected IFN-γ-treated L929 cell cultures. The added rickettsiae must be viable in order for macrophage death to occur [19], and macrophage death does not require tumor necrosis factor-α (TNF-α), is not dependent on nitric oxide synthase [23], and does not require the macrophage respiratory burst [60]. The exact mechanisms responsible for this macrophage death have not been defined.

Interestingly, although IFN-γ-pretreated macrophage-like RAW264.7 cells are damaged after *R. prowazekii* infection, the rickettsiae themselves retain their infectivity for (and ability to grow within) cultured Vero cells, and are not killed as the macrophages die [56]. Thus, host cell damage could represent a means for the rickettsiae to escape from the IFN-γ-treated host cells.

Both nitric oxide synthase-dependent and -independent mechanisms contribute to the antirickettsial effects of IFN-γ and TNF-α in cultured L929 cells [20,21,22,24]. However, the premature cellular injury observed in *R. prowazekii*-infected L929 cells that are treated with IFN-γ, TNF-α, or both cytokines is not dependent on nitric oxide synthase [22]. Increased phospholipid hydrolysis has been observed in *R. prowazekii*-infected L929 cells that are subsequently treated with IFN-γ [61]; this finding suggests possible involvement of a phospholipase.

The present study showed that IFN-γ-pretreated murine C166 endothelial cells were rapidly damaged after infection with *R. prowazekii*. Within several hours after addition of the rickettsiae, substantial numbers of the cells became permeable to trypan blue dye, were stained with ethidium bromide, and released LDH into the culture medium. These characteristics were not observed in the untreated infected cultures or in any of the mock-infected cultures, whether untreated or IFN-γ-pretreated. Previous studies have shown that IFN-γ-pretreated mouse macrophage-like RAW264.7 cells are rapidly damaged (as judged by their permeability to trypan blue dye) after infection with *R. prowazekii* [19,20,21,23]. The results with the IFN-γ-pretreated endothelial cells in the present study are similar to these earlier findings with IFN-γ-pretreated RAW264.7 cells. These results suggest that the endothelial cells are more similar to the macrophage-like cells (rather than the fibroblastic L929 cells) in their responses to IFN-γ treatment and *R. prowazekii* infection.

Next, the hypothesis that pore formation and osmotic lysis contribute to the rapid demise of the IFN-γ-pretreated endothelial cells after *R. prowazekii* infection was examined in the present study. In these experiments, PEGs of different molecular weights were tested for their ability to prevent the release of LDH, and the cells were stained with ethidium bromide. The rationale for this approach comes from previous studies [50,51], and is essentially that PEGs should be able to prevent the endothelial cells from releasing LDH into the culture medium if the PEGs are large enough to be excluded from the inside of the initially damaged endothelial cells. The experimental results showed that PEG 8000 strongly suppressed the release of LDH, PEG 4000 partially blocked the release of LDH, and PEGs 2000 and 1450 were ineffective at preventing the release of LDH. Although PEG 8000 prevented the release of LDH, it did not prevent the uptake of ethidium bromide by the IFN-γ-pretreated *R. prowazekii*-infected endothelial cells. These results suggest that pore formation and osmotic lysis are indeed involved in the observed injury to the endothelial cells. Based on the hydrodynamic radii of the PEGs, it can be estimated that the diameters of the pores are greater than 2.82 nm and less than 5.66 nm.

Earlier studies on the ability of different bacteria to kill macrophages used PEGs to check for the possible roles of pore formation and osmotic lysis in macrophage death [53,54]. These studies focused on macrophage death via pyroptosis after infection with *Salmonella enterica* serotype Typhimurium [53] or *Burkholderia pseudomallei* [54]. The macrophage death in each case was dependent on caspase-1, required a bacterial type III secretion system, was prevented by cytochalasin D (which blocked internalization of the bacteria), was associated with the release of interleukin-1β and interleukin-18, and involved pore formation and osmotic lysis. The estimated sizes of the membrane pores in these studies were smaller than those observed in the present study. For example, in the research with *S. enterica* Typhimurium, the membrane pores were estimated to be between 1.1 and 2.4 nm in diameter [53], while in the study with *B. pseudomallei*, the membrane pores were estimated to be smaller than (or close to) 3.84 nm in diameter [54]. The pore sizes in these studies likely reflect the pore sizes associated with the initial cellular damage caused by the bacteria used. It is now known that formation of gasdermin D pores occurs in association with pyroptosis; however, these pores are larger, as reviewed in [36,46], and the PEGs used in these earlier studies [53,54] (as well as in the present study) would probably have been too small to protect cells with these larger pores. It is important to remember that endothelial cells were used in the present study rather than macrophages. Nevertheless, pyroptosis has been observed in macrophages as well as other cell types (including intestinal epithelial cells and keratinocytes) [46], and it may occur in endothelial cells as well [62,63]. In this regard, it is interesting that endothelial cell death that is partially relieved by inhibition of caspase-1 has been observed in endothelial cells infected with a highly pathogenic *R. conorii* strain [43].

In another study, the possible involvement of pore formation and osmotic lysis was evaluated in the killing of macrophages by *Brucella abortus* rough mutants; this study reported that macrophage death was due to necrosis and oncosis [52]. Features of oncosis include cellular swelling as well as swelling of organelles [30]. In their experiments with macrophages and *B. abortus*, the researchers found that PEG 3350 partially inhibited macrophage release of LDH, whereas PEG 8000 was strongly inhibitory [52]. They suggested that perhaps a *B. abortus* type IV secretion system was required for macrophage injury, and a later study indeed demonstrated this requirement [64]. Interestingly, these results with PEGs [52] are similar to the findings in the present study with IFN-γ-pretreated endothelial cells and *R. prowazekii*. Here, PEG 4000 partially suppressed the release of LDH by IFN-γ-pretreated *R. prowazekii*-infected endothelial cells, whereas PEG 8000 dramatically suppressed it.

Exactly how the IFN-γ-pretreated endothelial cells in the present study were damaged by *R. prowazekii* is unknown. However, the finding that the cellular injury involves pore formation and osmotic lysis, along with an estimate of the pore size, provides a foundation for more specific future investigations. Pores or channels are involved in cell death that occurs by pyroptosis or necroptosis [44,45,46]. In addition, IFN-γ has been associated with enhancement of pyroptosis in macrophage-like RAW264.7 cells [65] as well as with increased necroptosis in lung epithelial cells (although not lung endothelial cells) [66].

Examination of the possible involvement of various caspases may help to clarify the mechanisms of cellular injury [28,29,30]. Perhaps a rickettsial secretion system (such as the type IV secretion system), a rickettsial effector(s), and/or even a pore-forming rickettsial effector [67,68] could play roles in the endothelial cell injury described in the current study. Rickettsial proteins that have already been associated with membrane lysis (TlyA, TlyC, Pat1, Pat2, and Pld) are of particular interest, and are reviewed in [4,5]. For example, the Pat 2 proteins, which are found only in certain *Rickettsia* species, are homologs of the *Pseudomonas aeruginosa* ExoU protein (a phospholipase A_2_) [69,70,71], and the phospholipase A activities of the *R. prowazekii* and *R. typhi* Pat2 proteins have been characterized [69,70]. It is interesting that researchers studying *P. aeruginosa* found that its ExoU protein activates caspase-1 during *P. aeruginosa* infection of cultured rat lung endothelial cells and injures the endothelial cells [72]. Additional research on *R. prowazekii*, as well as on other *Rickettsia* species, will likely reveal interesting details about how these obligate intracellular bacteria damage their host cells.

## Figures and Tables

**Table 1:** Effect of PEGs on *R. prowazekii*-induced release of LDH by interferon-treated C166 cells ^a^.

Treatment ^b^	Infection ^c^	Addition ^d^	% Total LDH Released at the following Time after Infection or Mock Infection				
			Removed ^e^	1 h	2 h	3 h	4 h
Untreated	Mock	No addition	—	0.2 ± 0.2 (10)	0.4 ± 0.4 (6)	0.4 ± 0.6 (16)	0.7 ± 0.5 (15)
		PEG 1450	—	0.0 ± 0.0 (2)	0.0 ± 0.0 (2)	0.0 ± 0.0 (2)	0.2 ± 0.2 (2)
		PEG 2000	—	0.0 ± 0.0 (2)	0.0 ± 0.0 (2)	0.0 ± 0.0 (2)	0.4 ± 0.4 (4)
		PEG 4000	—	1.3 ± 1.5 (6)	1.7 ± 0.9 (6)	1.5 ± 1.0 (6)	1.2 ± 1.0 (8)
		PEG 4000 A-1 h	—	—	0.5 ± 0.0 (2)	0.4 ± 0.0 (2)	0.4 ± 0.1 (2)
		PEG 8000	—	2.2 ± 0.6 (2)	1.7 ± 0.4 (2)	1.8 ± 0.5 (14)	2.1 ± 0.7 (12)
		PEG 8000 A-0.25 h	0.4 ± 0.1 (2)	—	—	1.2 ± 0.1 (2)	1.5 ± 0.1 (4)
		PEG 8000 A-1 h	—	—	—	—	1.7 ± 0.2 (4)
Interferon	Mock	No addition	—	0.4 ± 0.5 (10)	0.4 ± 0.7 (6)	0.6 ± 0.6 (16)	0.7 ± 0.6 (15)
		PEG 1450	—	0.0 ± 0.0 (2)	0.0 ± 0.0 (2)	0.0 ± 0.0 (2)	0.0 ± 0.0 (2)
		PEG 2000	—	0.0 ± 0.0 (2)	0.0 ± 0.0 (2)	0.0 ± 0.0 (2)	0.5 ± 0.7 (4)
		PEG 4000	—	1.2 ± 1.6 (6)	2.1 ± 1.7 (6)	4.1 ± 1.1 (6)	4.8 ± 2.5 (7)
		PEG 4000 A-1 h	—	—	0.3 ± 0.0 (2)	0.6 ± 0.1 (2)	0.6 ± 0.1 (2)
		PEG 8000	—	2.3 ± 0.4 (4)	2.3 ± 0.3 (4)	2.0 ± 0.5 (16)	2.7 ± 1.2 (14)
		PEG 8000 A-0.25 h	0.7 ± 0.5 (2)	—	—	1.6 ± 0.1 (2)	1.5 ± 0.2 (2)
		PEG 8000 A-1 h	—	—	—	—	1.6 ± 0.3 (4)
Untreated	Rp	No addition	—	0.3 ± 0.4 (11)	0.6 ± 0.4 (6)	0.6 ± 0.7 (16)	0.8 ± 0.4 (13)
		PEG 1450	—	0.0 ± 0.0 (2)	0.0 ± 0.0 (2)	0.0 ± 0.0 (2)	0.0 ± 0.0 (2)
		PEG 2000	—	0.0 ± 0.0 (2)	0.3 ± 0.4 (2)	0.3 ± 0.4 (2)	0.0 ± 0.0 (2)
		PEG 4000	—	0.8 ± 1.0 (5)	1.2 ± 1.1 (5)	1.6 ± 1.2 (5)	1.6 ± 1.1 (5)
		PEG 4000 A-1 h	—	—	0.4 ± 0.0 (2)	0.3 ± 0.2 (2)	0.3 ± 0.2 (2)
		PEG 8000	—	1.7 ± 0.2 (2)	2.8 ± 0.4 (2)	1.7 ± 0.5 (14)	2.6 ± 1.0 (12)
		PEG 8000 A-0.25 h	0.5 ± 0.2 (3)	—	—	1.3 ± 0.1 (2)	1.5 ± 0.2 (3)
		PEG 8000 A-1 h	—	—	—	—	1.5 ± 0.2 (3)
Interferon	Rp	No addition	—	3.9 ± 2.9 (11)	41.9 ± 3.8 (6)	55.2 ± 5.2 (16)	58.1 ± 8.8 (13)
		PEG 1450	—	2.7 ± 1.1 (4)	37.2 ± 3.2 (4)	52.3 ± 5.0 (4)	59.8 ± 10.0 (4)
		PEG 2000	—	2.9 ± 2.0 (4)	44.5 ± 0.9 (4)	59.1 ± 8.0 (4)	65.7 ± 14.1 (4)
		PEG 4000	—	1.0 ± 1.2 (6)	18.1 ± 2.4 (6)	30.9 ± 2.1 (6)	35.9 ± 4.7 (6)
		PEG 4000 A-1 h	—	—	16.9 ± 2.1 (2)	23.5 ± 2.7 (2)	26.0 ± 3.4 (2)
		PEG 8000	—	2.3 ± 0.4 (4)	3.2 ± 0.8 (4)	4.6 ± 1.0 (16)	5.3 ± 1.7 (15)
		PEG 8000 A-0.25 h	0.5 ± 0.2 (3)	—	—	4.8 ± 0.8 (2)	2.8 ± 0.2 (3)
		PEG 8000 A-1 h	—	—	—	—	3.0 ± 0.2 (3)

^a^ PEGs, polyethylene glycols; LDH, lactate dehydrogenase. Each value represents the mean ± standard deviation; the number of determinations is shown in parentheses. ^b^ Certain C166 cells were treated with gamma interferon (25 units/mL) for 22 to 25 h. ^c^ After treatment, all cells were washed and mock-infected (Mock) or infected with *Rickettsia prowazekii* Madrid E (Rp). ^d^ Unless otherwise noted, PEGs were present both during and after infection or mock infection. The letter “A” indicates that PEG was added after addition of the rickettsiae; the time of addition is shown after the “A”. In these instances, the rickettsiae or incubation medium was removed and replaced with fresh medium containing the appropriate PEG. All PEGs were used at a concentration of 30 mM, except for PEG 8000, which was used at a concentration of 15 mM due to its more limited solubility. ^e^ In certain instances, the rickettsiae or incubation medium that was removed from the cultures at 0.25 h after addition of the rickettsiae was later assayed for LDH. A result of — means not determined.

## Data Availability

Data supporting the results of this study may be found in the paper and in the Supplementary Materials.

## Supplementary Materials

The following supporting information can be downloaded at: https://www.mdpi.com/article/10.3390/tropicalmed7080163/s1. Figure S1: Results of ethidium bromide staining in experiment 77 (mock-infected), Results of ethidium bromide staining in experiment 77 (infected), Figure S1 legend; Figure S2: Results of ethidium bromide staining in experiment 79 (mock-infected), Results of ethidium bromide staining in experiment 79 (infected), Figure S2 legend; Figure S3: Results of ethidium bromide staining in experiment 72; Table S1: Data for Figure 1; Table S2: Rickettsial infection data for Figure 1; Table S3: Data for Figure 3; Table S4: LDH release data for Figure 3, Table 1 and Table S6; Table S5: Rickettsial infection data for Figure 3; Table S6: LDH release with lower PEG concentrations; Table S7: LDH release after removal of PEG.
