# Supplementary material for: Involvement of Pore Formation and Osmotic Lysis in the Rapid Killing of Gamma Interferon-Pretreated C166 Endothelial Cells by Rickettsia prowazekii
Source: Trop Med Infect Dis. 2022 Aug 1;7(8):163. doi: 10.3390/tropicalmed7080163 (PMC9415803; doi:10.3390/tropicalmed7080163)
Supplement: Supplementary file 1 [file tropicalmed-07-00163-s001.zip › tropicalmed-1822686-supplementary/Figure S2 legend.pdf]

Figure S2 legend

Figure S2. Results of ethidium bromide staining in experiment 79. This figure has two panels (Exp 79 Mock-infected and Exp 79 Infected), which are organized in separate files. Some images in Figure S2 are shown in Figure 5. In addition, this experiment (79) is a replicate of the experiment shown in Figure S1 (experiment 77). Ethidium bromide staining was done 3 to 6 hours after the start of the mock infection (Exp 79 Mock-infected) or 3 to 6 hours after addition of the rickettsiae (Exp 79 Infected).

For the Exp 79 Mock-infected panel, rows 1 and 5, untreated cultures without PEG; rows 2 and 6, untreated cultures with PEG 8000 d/a (PEG 8000 was added at the start of the mock-infection and was continuously present thereafter); rows 3 and 7, untreated cultures with PEG 8000 a15 (PEG 8000 was added after the 15-minute centrifugation); rows 4 and 8, untreated cultures with PEG a60 (PEG 8000 was added 60 minutes after the start of the mock-infection); rows 9 and 13, IFN-γ-pretreated cultures without PEG; rows 10 and 14, IFN-γ-pretreated cultures with PEG 8000 d/a; rows 11 and 15, IFN-γ-pretreated cultures with PEG 8000 a15; rows 12 and 16, IFN-γ-pretreated cultures with PEG 8000 a60.

For the Exp 79 Infected panel, rows 1 and 5, untreated cultures without PEG; rows 2 and 6, untreated cultures with PEG 8000 d/a (PEG 8000 was added along with the rickettsiae and was continuously present thereafter); rows 3 and 7, untreated cultures with PEG 8000 a15 (PEG 8000 was added after the 15-minute centrifugation); rows 4 and 8, untreated cultures with PEG a60 (PEG 8000 was added 60 minutes after addition of the rickettsiae); rows 9 and 13, IFN-γ-pretreated cultures without PEG; rows 10 and 14, IFN-γ-pretreated cultures with PEG 8000 d/a (PEG 8000 was added along with the rickettsiae and was continuously present thereafter); rows 11 and 15, IFN-γ-pretreated cultures with PEG 8000 a15 (PEG 8000 was added after the 15-minute centrifugation); rows 12 and 16, IFN-γ-pretreated cultures with PEG 8000 a60 (PEG 8000 was added 60 minutes after addition of the rickettsiae).

All transmitted light images in the A columns were captured under identical conditions, and the same adjustments for exposure and contrast were applied to each, entire image using Adobe Photoshop CC 2018® software. All fluorescence images shown in the B columns were captured under identical conditions and no adjustments were made. In the C columns (Fluorescence*), the images from the B columns were adjusted for exposure and contrast using the same settings applied to the images in the A columns; Adobe Photoshop CC 2018® software was used. In the D columns, the overlay images were adjusted in the same manner as the images in the A columns.

The column designations (A through D) and row labels (1 through 16) were used below to identify the images included in Figure 5. The images in each panel of Figure 5 (A through H), are listed in order of occurrence, beginning with the top row, and proceeding left to right; and then continuing with the second row, from left to right, in a similar manner; and so on. The images from the Figure S2 Exp 79 Mock-infected panel that are shown in Figure 5 Panel A are as follows: 1A, 1B, 1D; 2A, 2B, 2D; 3A, 3B, 3D; 4A, 4B, 4D. For Panel B of Figure 5, the images from the Figure S2 Exp 79 Mock-infected panel are 5A, 5B, 5D; 6A, 6B, 6D; 7A, 7B, 7D; 8A, 8B, 8D; for Panel C of Figure 5, the images are 9A, 9B, 9D; 10A, 10B, 10D; 11A, 11B, 11D, 12A, 12B, 12D; and for Panel D of Figure 5, the images are 13A, 13B, 13D; 14A, 14B, 14D; 15A, 15B, 15D; 16A, 16B, 16D. Similarly, the images from the Figure S2 Exp 79 Infected panel that are shown in Figure 5 Panel E are as follows: 1A, 1B, 1D; 2A, 2B, 2D; 3A, 3B, 3D; 4A, 4B, 4D. For Panel F of Figure 5, the images from the Figure S2 Exp 79 Infected panel are 5A, 5B, 5D; 6A, 6B, 6D; 7A, 7B, 7D; 8A, 8B, 8D; for Panel G of Figure 5, the images are 9A, 9B, 9D; 10A, 10B, 10D; 11A, 11B, 11D, 12A, 12B, 12D; and for Panel H of Figure 5, the images are 13A, 13B, 13D; 14A, 14B, 14D; 15A, 15B, 15D; 16A, 16B, 16D.
