# Supplementary material for: Involvement of Pore Formation and Osmotic Lysis in the Rapid Killing of Gamma Interferon-Pretreated C166 Endothelial Cells by Rickettsia prowazekii
Source: Trop Med Infect Dis. 2022 Aug 1;7(8):163. doi: 10.3390/tropicalmed7080163 (PMC9415803; doi:10.3390/tropicalmed7080163)
Supplement: Supplementary file 1 [file tropicalmed-07-00163-s001.zip › tropicalmed-1822686-supplementary/Figure S3, Results of ethidium bromide staining in experiment 72.pdf]

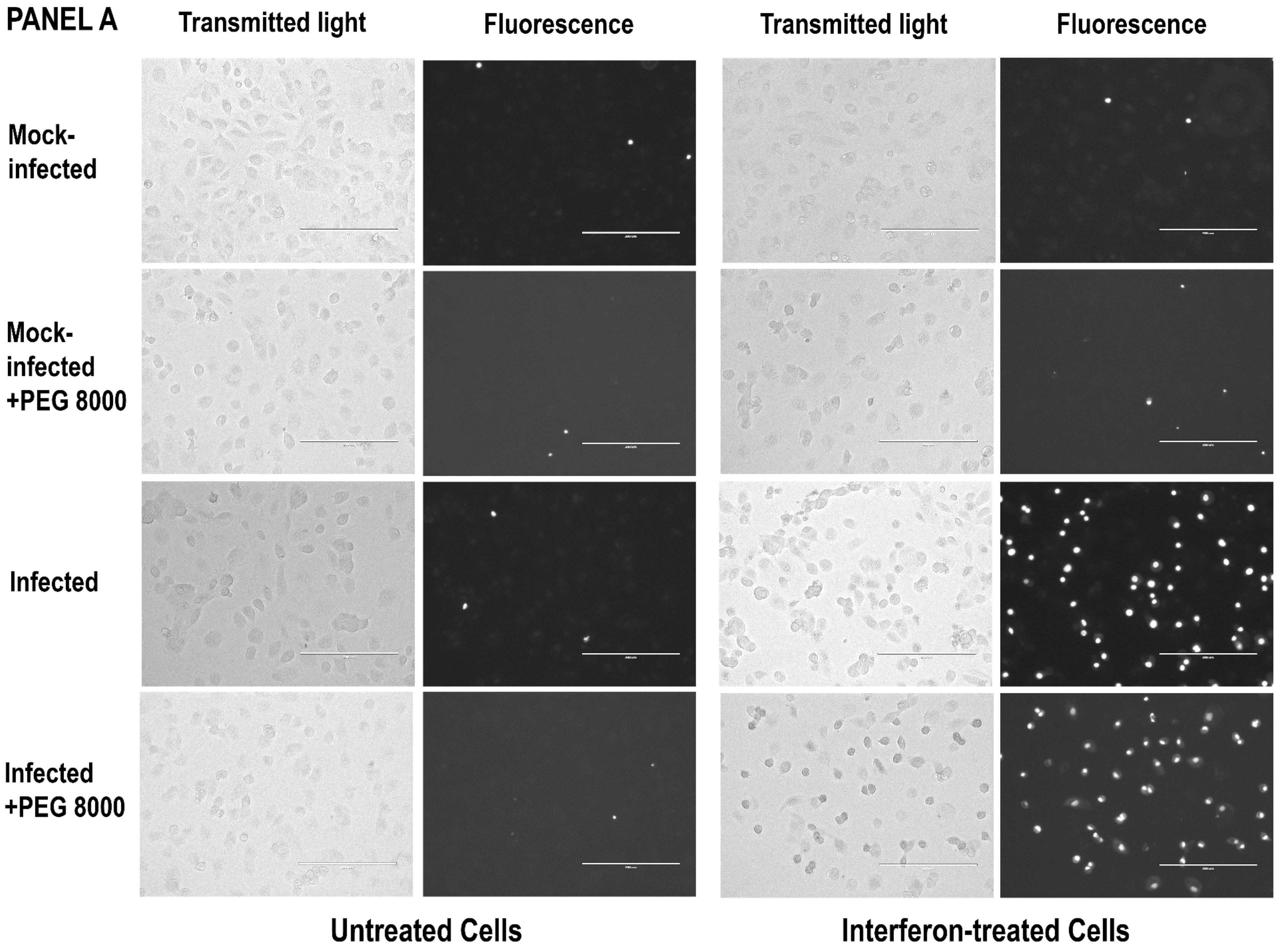
PANEL A
Transmitted light
Fluorescence
Transmitted light
Fluorescence
Mock-infected
Mock-infected +PEG 8000
Infected
Infected +PEG 8000
Untreated Cells
Interferon-treated Cells

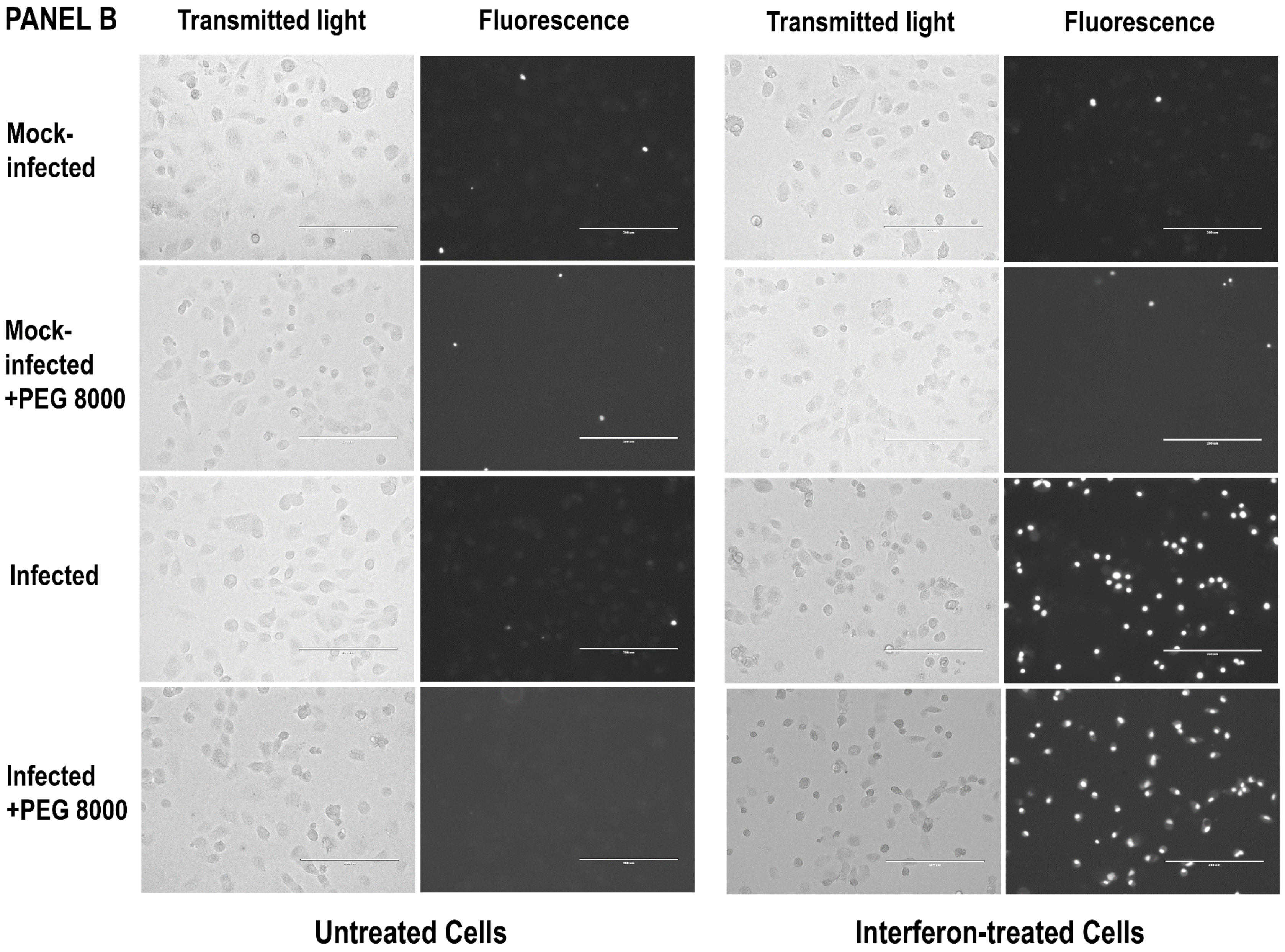
PANEL B
Transmitted light
Fluorescence
Transmitted light
Fluorescence
Mock-infected
Mock-infected +PEG 8000
Infected
Infected +PEG 8000
Untreated Cells
Interferon-treated Cells

Figure S3, Results of ethidium bromide staining in experiment 72. PEG 8000 did not prevent the IFN-γ-pretreated, *R. prowazekii*-infected endothelial cells from being stained with ethidium bromide, even though it suppressed the release LDH. This figure has two panels (A and B), which represent duplicate cultures in this experiment. PEG 8000 was added at the start of the mock-infection or along with the rickettsiae, and it was continuously present thereafter. Ethidium bromide staining was done at 3 to 6 hours after the start of the mock infection or addition of the rickettsiae. Each bar represents 200 µm. Each image was adjusted using Adobe Photoshop® software. In each case, the adjustments were applied to the entire image. However, different adjustments may have been applied to different images to optimize the appearance of the images.
