# Supplementary material for: Involvement of Pore Formation and Osmotic Lysis in the Rapid Killing of Gamma Interferon-Pretreated C166 Endothelial Cells by Rickettsia prowazekii
Source: Trop Med Infect Dis. 2022 Aug 1;7(8):163. doi: 10.3390/tropicalmed7080163 (PMC9415803; doi:10.3390/tropicalmed7080163)
Supplement: Supplementary file 1 [file tropicalmed-07-00163-s001.zip › tropicalmed-1822686-supplementary/Table S1 Data for Figure 1.pdf]

Table S1

Data for Figure 1 **Percent trypan blue-positive cells**

	RpE	RpE	Mock	Mock	Uninfected prep (1/10)	Uninfected prep (1/20)	Uninfected prep (1/10)	Uninfected prep (1/20)
Exp 43								
Exp44								
Exp 49	C166 C	C166 I	C166 C	C166 I	C166 Control	C166 Control	C166 IFN	C166 IFN
Expt 51	2.6	44.4	0.0	0.0	11.6	3.7	8.2	3.0
Exp53	7.6	39.1	1.0	0.0	8.7	3.4	11.5	3.8
Exp56	1.0	64.7	1.0	1.0	7.8		12.9	
Exp57	1.0	98.0	0.0	0.0				
Exp59	2.0	92.4	1.0	0.0				
Exp60	4.0	87.4	0.0	0.0				
Exp62	7.1	81.9	0.0	0.0				
Exp63	3.3	95.8	0.0	0.0				
Exp64	5.0	93.8	0.0	1.0				
	1.0	75.2	1.0	0.0				
	0.0	82.1	1.0	0.0				
	0.0	80.5	2.0	0.0				
	3.0	48.5	1.0	2.0				
	2.0	48.3	1.0	1.0				
	3.0	84.2	0.0	0.0				
	1.9	86.6	1.0	1.0				
	2.0	89.7	0.0	2.0				
	3.4	88.2	0.0	2.0				
	0.0	95.6	0.0	1.0				
	1.0	94.5		0.0				
	12.0	90.1		1.0				
	9.6	96.3						
	3.6	95.2						
	7.5	96.2						
		96.6						
		97.1						
Avg	3.5	82.4	0.5	0.6	9.4	3.6	10.9	3.4
Std Dev	3.2	18.0	0.6	0.7	2.0	0.2	2.4	0.6
	3.5	82.4	0.5	0.6	9.4	3.6	10.9	3.4
	3.2	18.0	0.6	0.7	2.0	0.2	2.4	0.6
	3.5	82.4	0.5	0.6	9.4	3.6	10.9	3.4
	3.2	18.0	0.6	0.7	2.0	0.2	2.4	0.6

C=Untreated control cultures
I=Interferon-treated cultures
M=Mock-infected
UP (uninfected prep)= lysate of uninfected L929 cells
RpE=*Rickettsia prowazekii* Madrid E
