# Supplementary material for: Involvement of Pore Formation and Osmotic Lysis in the Rapid Killing of Gamma Interferon-Pretreated C166 Endothelial Cells by Rickettsia prowazekii
Source: Trop Med Infect Dis. 2022 Aug 1;7(8):163. doi: 10.3390/tropicalmed7080163 (PMC9415803; doi:10.3390/tropicalmed7080163)
Supplement: Supplementary file 1 [file tropicalmed-07-00163-s001.zip › tropicalmed-1822686-supplementary/Table S2 Rickettsial Infection Data for Figure 1.pdf]

Table S2

Rickettsial Infection Data for Figure 1

	Dilution	Prep #		C+RpE %R	RI	NR*100	NR	I+RpE %R	RI	NR*100	NR
Exp 43	1/500	8		100	16.15	1615	16.15	99	21.98	2176	21.76
Exp 44	1/1000	8		94	8.99	845	8.45	96	10.69	1026	10.26
	1/1000	8		93	10.25	953	9.53	96	8.04	772	7.72
Exp 49	1/500	10		79	5.81	459	4.59	83	6.66	553	5.53
Exp 51	1/300	10		84	7.24	608	6.08	79	8.73	690	6.9
	1/300	10		89	11.19	996	9.96	94	11.24	1057	10.57
Exp 53	1/250	10		99	17.75	1757	17.57	100	28.04	2804	28.04
Exp 56	1/260	10		98	19.93	1953	19.53	100	18.13	1813	18.13
Exp 59	1/200	10		99	14.59	1444	14.44	98	13.46	1319	13.19
Exp 59	1/200	10		95	12.4	1178	11.78	100	15.4	1540	15.4
Exp 60	1/200	10		100	17.22	1722	17.22	100	19.36	1936	19.36
Exp 60	1/200	10		96	14.06	1350	13.5	99	16.36	1620	16.2
Exp 62	1/200	10		98	14.37	1408	14.08	100	15.61	1561	15.61
Exp 62	1/200	10		97	14.88	1443	14.33	99	14.62	1447	14.47
Exp 63	1/200	10		97	9.14	887	8.87	100	18.74	1874	18.74
Exp 63	1/200	10		100	23.77	2377	23.77	100	27.94	2794	27.94
Exp 64	1/200	10		not determined							
Exp 57	1/260	10		not determined							
			AVG	94.9	13.6	1312.2	13.1	96.4	15.9	1561.4	15.6
			STD DEV	6.1	4.8	511.0	5.1	6.3	6.4	671.2	6.7

%R= percent infected
RI=rickettsiae per infected cell
NR*100=rickettsiae in 100 cells
NR=rickettsiae per cell
C=Untreated Control cultures
I=Interferon-treated cultures
RpE=*Rickettsia prowazekii* Madrid E
