# Supplementary material for: Involvement of Pore Formation and Osmotic Lysis in the Rapid Killing of Gamma Interferon-Pretreated C166 Endothelial Cells by Rickettsia prowazekii
Source: Trop Med Infect Dis. 2022 Aug 1;7(8):163. doi: 10.3390/tropicalmed7080163 (PMC9415803; doi:10.3390/tropicalmed7080163)
Supplement: Supplementary file 1 [file tropicalmed-07-00163-s001.zip › tropicalmed-1822686-supplementary/Table S3 Data for Figure 3.pdf]

**Table S3** **Percentage of Total LDH Released**

**Data for Figure 3**

| Hours | Mk Ctrl | | RpL Ctrl | | RpM Ctrl | | RpH Ctrl | | Mk IFN | | RpL IFN | | RpM IFN | | RpH IFN | |
|---|---|---|---|---|---|---|---|---|---|---|---|---|---|---|---|---|
| | Mean | SD | Mean | SD | Mean | SD | Mean | SD | Mean | SD | Mean | SD | Mean | SD | Mean | SD |
| 1 | 0.2 | 0.2 | 0.0 | 0.0 | 0.3 | 0.4 | 0.1 | 0.2 | 0.4 | 0.5 | 1.6 | 0.4 | 3.9 | 2.9 | 6.6 | 1.2 |
| 2 | 0.4 | 0.4 | 0.5 | 0.1 | 0.6 | 0.4 | 0.2 | 0.3 | 0.4 | 0.7 | 28.4 | 1.4 | 41.9 | 3.8 | 55.4 | 1.2 |
| 3 | 0.4 | 0.6 | 1.6 | 0.7 | 0.6 | 0.7 | 1.3 | 0.2 | 0.6 | 0.6 | 39.4 | 2.1 | 55.2 | 5.2 | 64.7 | 3.8 |
| 4 | 0.7 | 0.5 | 1.0 | 0.2 | 0.8 | 0.4 | 1.4 | 0.1 | 0.7 | 0.6 | 44.0 | 1.3 | 58.1 | 8.8 | 72.4 | 7.0 |
| **# of determinations** | **10** | | **4** | | **11** | | **4** | | **10** | | **4** | | **11** | | **4** | |
| | **6** | | **2** | | **6** | | **2** | | **6** | | **2** | | **6** | | **2** | |
| | **16** | | **2** | | **16** | | **2** | | **16** | | **2** | | **16** | | **2** | |
| | **15** | | **2** | | **13** | | **4** | | **15** | | **2** | | **13** | | **4** | |

Mk Ctrl=Untreated, mock-infected
RpL Ctrl=Untreated, RpLow infected
RpM Ctrl=Untreated, RpMedium infected
RpH Ctrl=Untreated, RpHigh infected

Mk IFN=IFN-treated, mock-infected
RpL IFN=IFN-treated, RpLow infected
RpM IFN=IFN-treated, RpMedium infected
RpH IFN=IFN-treated, RpHigh infected

SD, standard deviation

LDH, lactate dehydrogenase
