# Supplementary material for: Involvement of Pore Formation and Osmotic Lysis in the Rapid Killing of Gamma Interferon-Pretreated C166 Endothelial Cells by Rickettsia prowazekii
Source: Trop Med Infect Dis. 2022 Aug 1;7(8):163. doi: 10.3390/tropicalmed7080163 (PMC9415803; doi:10.3390/tropicalmed7080163)
Supplement: Supplementary file 1 [file tropicalmed-07-00163-s001.zip › tropicalmed-1822686-supplementary/Table S4 LDH Release Data for Fig 3, Tables 1 and S6.pdf]

| | |
|---|---|
| **TABLE S4, SHEET 1 (pages 1-14) -- PERCENTAGE OF TOTAL LDH RELEASED** | **ABBREVIATIONS USED IN THIS TABLE ARE DEFINED ON PAGE 14** |
| **LDH Release Data for Fig 3, Table 1, & Table S6** | **TREATMENTS IN SHEET 1 (pages 1-14) ARE SHOWN BELOW IN DARK BLUE, BOLD-FACE TYPE** |

**The purpose of this table (Table S4) is to provide the individual values for the data (means and standard deviations) shown in Fig 3, Table 1, & Table S6**

**In this table (Table S4) each mean and standard deviation is shown below the corresponding individual values.**

**Some data shown here are summarized in Figure 3, Table 1 & Table S6.**

**Data (means & standard deviations) used in Figure 3 are highlighted in pink below.**

Sheet 2 gives an additional summary of some of the means & standard deviations in this table

**The numbers 1, 2, 3, & 4 indicate the hours after addition of the rickettsiae or mock-infection.**

| | | **1** | **2** | **3** | **4** |
|---|---|---|---|---|---|
| black=69 | **Mk Ctrl** | 0.03 | 1.07 | 1.32 | 1.28 |
| red=70 | | 0.00 | 0.6 | 0.85 | 0.84 |
| green=71 | | 0.2 | 0.5 | 0.2 | 0.7 |
| black=68 | | 0 | 0.1 | 0 | 0.3 |
| red=72 | | 0.4 | 0 | 0 | 2.1 |
| green=74 | | 0.3 | 0 | 0 | 0 |
| black=75 | | ***0.00*** | | 2.3 | 0.6 |
| purple=80 | | ***0.00*** | | 0.1 | 0.7 |
| ***black=69 later*** | | 0.5 | | 0 | 0.5 |
| | | 0.3 | | 0.1 | 0.8 |
| | | | | 0 | 0.5 |
| | | | | 0 | 0.6 |
| | | | | 0.3 | 0.4 |
| | | | | 0.2 | 0.7 |
| | | | | 0.5 | 0.2 |
| | | | | 0.2 | |
| **Mk Ctrl** | Mean | **0.18** | **0.38** | **0.38** | **0.68** |
| | Std Dev | **0.20** | **0.43** | **0.63** | **0.49** |

| | | **1** | **2** | **3** | **4** |
|---|---|---|---|---|---|
| **Mk Ctrl** | **Peg 4000 d/a** | 0.23 | 0.79 | 1.52 | 0.61 |
| | | 0 | 1.12 | 0.49 | 0.88 |
| | | 0.4 | 1.9 | 1.1 | 1.1 |
| | | 1.1 | 1.2 | 0.5 | 1.1 |
| | | 3.9 | 3.3 | 3.3 | 3.4 |
| | | 2.4 | 1.8 | 1.8 | 2 |
| | | | | | 0.5 |
| | | | | | 0.3 |
| | Mean | **1.3** | **1.7** | **1.5** | **1.2** |
| | Std Dev | **1.5** | **0.9** | **1.0** | **1.0** |

| | | **1** | **2** | **3** | **4** |
|---|---|---|---|---|---|
| | **RpL Ctrl** | 0 | 0.59 | 2.1 | 0.82 |
| | | 0 | 0.46 | 1.07 | 1.11 |
| | | ***0.00*** | | | |
| | | ***0.00*** | | | |
| **RpL Ctrl** | Mean | **0.00** | **0.53** | **1.59** | **0.97** |
| | Std Dev | **0.00** | **0.09** | **0.73** | **0.21** |

| | | **1** | **2** | **3** | **4** |
|---|---|---|---|---|---|
| **RpL Ctrl** | **Peg 4000 d/a** | 0 | 0.29 | 0.63 | 0.75 |
| | | 0 | 0.37 | 0.7 | 0.55 |
| | Mean | **0.0** | **0.3** | **0.7** | **0.7** |
| | Std Dev | **0.0** | **0.1** | **0.0** | **0.1** |

| | | | | |
|---|---|---|---|---|
| **RpM Ctrl** | 0 | 0.67 | 2.97 | 1.07 |
| | 0.08 | 0.59 | 1.19 | 0.79 |
| | 0.3 | 0.3 | 0.3 | 1.1 |
| | 0.4 | 1.1 | 0.2 | 0.9 |
| | 0 | 1.1 | 1.2 | 0 |
| | 0 | 0 | 0 | 0 |
| | ***0.00*** | | 0.5 | 0.8 |
| | ***0.00*** | | 0.4 | 1 |
| | 0.9 | | 0.6 | 1.1 |
| | 0.4 | | 0.4 | 1 |
| | 0.9 | | 0.1 | 1.1 |
| | | | 0.2 | 0.5 |
| | | | 0.1 | 0.4 |
| | | | 0.3 | |
| | | | 0.3 | |
| | | | 0.4 | |

| | | | | | |
|---|---|---|---|---|---|
| **RpM Ctrl** | Mean | **0.28** | **0.63** | **0.57** | **0.75** |
| | Std Dev | **0.36** | **0.44** | **0.73** | **0.40** |

| | | | | | |
|---|---|---|---|---|---|
| **RpM Ctrl** | **Peg 4000 d/a** | 0 | 1.03 | 0.89 | 1.25 |
| | | 0.02 | 0.38 | 0.65 | 0.72 |
| | | 0.5 | 1.1 | 1.1 | 1.1 |
| | | 0.8 | 0.6 | 1.9 | 1.4 |
| | | 2.5 | 3.1 | 3.6 | 3.6 |
| | Mean | **0.8** | **1.2** | **1.6** | **1.6** |
| | Std Dev | **1.0** | **1.1** | **1.2** | **1.1** |

| | | | | |
|---|---|---|---|---|
| **RpH Ctrl** | 0 | 0 | 1.17 | 1.35 |
| | 0.07 | 0.42 | 1.42 | 1.52 |
| | ***0.35*** | | | 1.4 |
| | ***0.00*** | | | 1.3 |

| | | | | | |
|---|---|---|---|---|---|
| **RpH Ctrl** | Mean | **0.11** | **0.21** | **1.30** | **1.39** |
| | Std Dev | **0.17** | **0.30** | **0.18** | **0.09** |

| | | | | | |
|---|---|---|---|---|---|
| **RpH Ctrl** | **Peg 4000 d/a** | 0 | 0.48 | 0.7 | 0.78 |
| | | 0 | 0.39 | 0.61 | 0.67 |
| | | | | | 0.9 |
| | | | | | 1.3 |
| | Mean | **0.0** | **0.4** | **0.7** | **0.9** |
| | Std Dev | **0.0** | **0.1** | **0.1** | **0.3** |

| | | | | |
|---|---|---|---|---|
| **Mk IFN** | 0.05 | 1.88 | 2.22 | 1.92 |
| | 0 | 0 | 1.04 | 0.82 |
| | 0.4 | 0.4 | 0.9 | 1.8 |
| | 0.1 | 0.4 | 0.2 | 0.3 |
| | 0.4 | 0 | 0 | 0 |
| | 1.6 | 0 | 1.1 | 0 |
| | ***0.00*** | | 0.7 | 0.9 |
| | ***0.00*** | | 0.5 | 1 |
| | 0.4 | | 0.6 | 0.6 |
| | 1.0 | | 0.5 | 0.3 |
| | | | 0.3 | 0.6 |
| | | | 0.9 | 0.6 |
| | | | 0.1 | 0.9 |
| | | | 0.1 | 0.4 |
| | | | 0.2 | 0.7 |
| | | | 0.1 | |

| | | | | | |
|---|---|---|---|---|---|
| **Mk IFN** | Mean | **0.40** | **0.45** | **0.59** | **0.72** |
| | Std Dev | **0.53** | **0.73** | **0.57** | **0.55** |

| | | | | | | |
|---|---|---|---|---|---|---|
| **Mk IFN** | **Peg 4000 d/a** | 0.11 | 0 | 2.51 | 2.95 | 2.95 |
| | | 0.02 | 0 | 3.07 | | 8 |
| | | 1 | 3.1 | 5.1 | 8 | 7.7 |
| | | 1.5 | 3.1 | 5.4 | 7.7 | 5.6 |
| | | 0.4 | 3 | 4.1 | 5.6 | 5.5 |
| | | 4.3 | 3.6 | 4.2 | 5.5 | 1.9 |
| | | | | | 1.9 | 2.2 |
| | | | | | 2.2 | |
| | Mean | **1.2** | **2.1** | **4.1** | **4.8** | **4.8** |
| | Std Dev | **1.6** | **1.7** | **1.1** | **2.5** | **2.5** |

| | | | | | |
|---|---|---|---|---|---|
| RpL IFN | | 1.57 | 27.4 | 37.84 | 43.07 |
| | | 1.6 | 29.45 | 40.87 | 44.96 |
| | | ***1.08*** | | | |
| | | ***2.04*** | | | |
| RpL IFN | Mean | **1.57** | **28.43** | **39.36** | **44.02** |
| | Std Dev | **0.39** | **1.45** | **2.14** | **1.34** |

| | | | | | |
|---|---|---|---|---|---|
| RpL IFN | Peg 4000 d/a | 0 | 12.62 | 21.88 | 27.74 |
| | | 0.48 | 11.27 | 21.26 | 23.35 |
| | Mean | **0.2** | **11.9** | **21.6** | **25.5** |
| | Std Dev | **0.3** | **1.0** | **0.4** | **3.1** |

| | | | | | |
|---|---|---|---|---|---|
| RpM IFN | | 3.76 | 41.75 | 52.12 | 55.62 |
| | | 2.81 | 43.3 | 54.58 | 58.46 |
| | | 5 | 43.3 | 57.1 | 65.4 |
| | | 3.9 | 47.5 | 63 | 74.3 |
| | | 8.9 | 38.6 | 47.5 | 49.1 |
| | | 9.5 | 36.9 | 43.8 | 46.1 |
| | | ***2.26*** | | 60.3 | 66.1 |
| | | ***3.02*** | | 58 | 61.4 |
| | | 1.3 | | 61.5 | 67.5 |
| | | 1.2 | | 58.6 | 62.1 |
| | | 1.3 | | 57.2 | 50 |
| | | | | 59.9 | 49.4 |
| | | | | 50.8 | 50 |
| | | | | 53.1 | |
| | | | | 53.2 | |
| | | | | 52.4 | |
| RpM IFN | Mean | **3.91** | **41.89** | **55.19** | **58.11** |
| | Std Dev | **2.88** | **3.77** | **5.23** | **8.82** |

| | | | | | |
|---|---|---|---|---|---|
| RpM IFN | Peg 4000 d/a | 0.04 | 18.57 | 29.4 | 32.04 |
| | | 0 | 18.89 | 29.89 | 34.25 |
| | | 1 | 15.7 | 34.2 | 40.6 |
| | | 0.4 | 14.7 | 32.7 | 43.1 |
| | | 3.1 | 20.7 | 30.3 | 33.4 |
| | | 1.5 | 20.1 | 28.9 | 32.2 |
| | Mean | **1.0** | **18.1** | **30.9** | **35.9** |
| | Std Dev | **1.2** | **2.4** | **2.1** | **4.7** |

| | | | | | |
|---|---|---|---|---|---|
| RpH IFN | | 7.78 | 54.58 | 62.02 | 64.64 |
| | | 6.68 | 56.31 | 67.41 | 68.42 |
| | | ***5.04*** | | | 78.1 |
| | | ***6.94*** | | | 78.6 |
| RpH IFN | Mean | **6.61** | **55.45** | **64.72** | **72.44** |
| | Std Dev | **1.15** | **1.22** | **3.81** | **7.00** |

| | | | | | |
|---|---|---|---|---|---|
| RpH IFN | Peg 4000 d/a | 0.12 | 28.44 | 35.54 | 36.94 |
| | | 0.32 | 27.08 | 35.05 | 36.74 |
| | | | | | 30.3 |
| | | | | | 30.4 |
| | Mean | **0.2** | **27.8** | **35.3** | **33.6** |
| | Std Dev | **0.1** | **1.0** | **0.3** | **3.7** |

| | | | | | |
|---|---|---|---|---|---|
| Mk Ctrl | Peg 8000 d/a | 2.6 | 1.9 | 2.8 | 2.7 |
| | | 1.7 | 1.4 | 1.4 | 1.6 |
| | | | | 1.8 | 1.8 |
| | | | | 2.6 | 1.7 |
| | | | | 2.1 | 1.8 |
| | | | | 1.6 | 2.8 |
| | | | | 2.1 | 2.2 |
| | | | | 1.2 | 3.6 |
| | | | | 1.4 | 2.9 |
| | | | | 1.3 | 1.5 |
| | | | | 1.5 | 1.6 |
| | | | | 1.3 | 1.4 |
| | | | | 2.1 | |
| | | | | 1.3 | |
| | Mean | **2.2** | **1.7** | **1.8** | **2.1** |
| | Std Dev | **0.6** | **0.4** | **0.5** | **0.7** |

| | | | | | |
|---|---|---|---|---|---|
| Mk Ctrl | Peg 2000 d/a | 0 | 0 | 0 | 0 |
| | | 0 | 0 | 0 | 0 |
| | | | | | 0.6 |
| | | | | | 0.8 |
| | Mean | 0.0 | 0.0 | 0.0 | 0.4 |
| | Std Dev | 0.0 | 0.0 | 0.0 | 0.4 |
| Mk Ctrl | Peg 4000-15d/a | | | | 1 |
| | | | | | 1 |
| | Mean | | | | 1.0 |
| | Std Dev | | | | 0.0 |
| Mk Ctrl | Peg 1450 d/a | 0 | 0 | 0 | 0.3 |
| | | 0 | 0 | 0 | 0 |
| | Mean | 0.0 | 0.0 | 0.0 | 0.2 |
| | Std Dev | 0.0 | 0.0 | 0.0 | 0.2 |
| Mk Ctrl | Peg 400 d/a | | | | |
| RpM Ctrl | Peg 8000 d/a | 1.8 | 3 | 2.7 | 3 |
| | | 1.5 | 2.5 | 2.3 | 2.2 |
| | | | | 1.6 | 1.8 |
| | | | | 1.8 | 2.9 |
| | | | | 0.9 | 4.8 |
| | | | | 1.8 | 2.1 |
| | | | | 1.7 | 2.4 |
| | | | | 1 | 4.2 |
| | | | | 1.7 | 2.4 |
| | | | | 1.6 | 2.5 |
| | | | | 1.7 | 1.4 |
| | | | | 1.3 | 1.4 |
| | | | | 1.7 | |
| | | | | 2.1 | |
| | Mean | 1.7 | 2.8 | 1.7 | 2.6 |
| | Std Dev | 0.2 | 0.4 | 0.5 | 1.0 |
| RpM Ctrl | Peg 2000 d/a | 0 | 0.5 | 0 | 0 |
| | | 0 | 0 | 0.5 | 0 |
| | Mean | 0.0 | 0.3 | 0.3 | 0.0 |
| | Std Dev | 0.0 | 0.4 | 0.4 | 0.0 |

| | | | | | |
|---|---|---|---|---|---|
| **RpM Ctrl** | **Peg 1450 d/a** | 0 | 0 | 0 | 0 |
| | | 0 | 0 | 0 | 0 |
| | Mean | **0.0** | **0.0** | **0.0** | **0.0** |
| | Std Dev | **0.0** | **0.0** | **0.0** | **0.0** |
| **RpM Ctrl** | **Peg 400 d/a** | 0 | 0 | 0 | 0 |
| | | 0 | 0 | 0 | 0 |
| | Mean | **0.0** | **0.0** | **0.0** | **0.0** |
| | Std Dev | **0.0** | **0.0** | **0.0** | **0.0** |
| **Mk IFN** | **Peg 8000 d/a** | 2.3 | 2.2 | 2.3 | 2.6 |
| | | 2.8 | 2.7 | 2.5 | 3 |
| | | 2.3 | 1.9 | 1.5 | 1.6 |
| | | 1.9 | 2.4 | 1.8 | 1.9 |
| | | | | 2.2 | 6 |
| | | | | 2.8 | 2.6 |
| | | | | 2.8 | 2.6 |
| | | | | 2.2 | 3.7 |
| | | | | 1.3 | 3.5 |
| | | | | 1.6 | 3.3 |
| | | | | 1.6 | 1.8 |
| | | | | 1.6 | 2.3 |
| | | | | 1.6 | 1.6 |
| | | | | 1.6 | 1.8 |
| | | | | 2.2 | |
| | | | | 2.5 | |
| | Mean | **2.3** | **2.3** | **2.0** | **2.7** |
| | Std Dev | **0.4** | **0.3** | **0.5** | **1.2** |
| **Mk IFN** | **Peg 2000 d/a** | 0 | 0 | 0 | 0 |
| | | 0 | 0 | 0 | 0 |
| | | | | | 0.6 |
| | | | | | 1.4 |
| | Mean | **0.0** | **0.0** | **0.0** | **0.5** |
| | Std Dev | **0.0** | **0.0** | **0.0** | **0.7** |
| **Mk IFN** | **Peg 4000-15d/a** | | | | 1.2 |
| | | | | | 1.2 |
| | Mean | | | | **1.2** |
| | Std Dev | | | | **0.0** |

| | | | | | |
|---|---|---|---|---|---|
| Mk IFN | Peg 1450 d/a | 0 | 0 | 0 | 0 |
| | | 0 | 0 | 0 | 0 |
| | Mean | **0.0** | **0.0** | **0.0** | **0.0** |
| | Std Dev | **0.0** | **0.0** | **0.0** | **0.0** |

| | | | | | |
|---|---|---|---|---|---|
| Mk IFN | Peg 400 d/a | | | | |

| | | | | | |
|---|---|---|---|---|---|
| RpM IFN | Peg 8000 d/a | 2.8 | 4.1 | 3.3 | 4.8 |
| | | 2.2 | 3.6 | 3.2 | 3.9 |
| | | 2 | 2.5 | 3 | 3.5 |
| | | 2 | 2.5 | 3.3 | 4.3 |
| | | | | 3.9 | 4.7 |
| | | | | 5.1 | 5.7 |
| | | | | 5.5 | 5.1 |
| | | | | 3.9 | 5.2 |
| | | | | 4.6 | 8.5 |
| | | | | 4.7 | 7.3 |
| | | | | 5.2 | 5.7 |
| | | | | 5 | 7.3 |
| | | | | 5.1 | 3.4 |
| | | | | 5.8 | 2.9 |
| | | | | 6.5 | 3 |
| | | | | 4.9 | |
| | Mean | **2.3** | **3.2** | **4.6** | **5.0** |
| | Std Dev | **0.4** | **0.8** | **1.0** | **1.7** |

| | | | | | |
|---|---|---|---|---|---|
| RpM IFN | Peg 2000 d/a | 1.5 | 45.7 | 65.3 | 78.3 |
| | | 1.4 | 44.3 | 66.6 | 77.5 |
| | | 5.7 | 44.3 | 51.5 | 53.6 |
| | | 3.1 | 43.6 | 53 | 53.5 |
| | Mean | **2.9** | **44.5** | **59.1** | **65.7** |
| | Std Dev | **2.0** | **0.9** | **8.0** | **14.1** |

| | | | | | |
|---|---|---|---|---|---|
| RpM IFN | Peg 1450 d/a | 1.8 | 37.7 | 59.1 | 73.1 |
| | | 2.1 | 32.8 | 53.1 | 61.9 |
| | | 2.8 | 37.7 | 47.9 | 51.1 |
| | | 4.2 | 40.6 | 49.1 | 53.2 |
| | Mean | **2.7** | **37.2** | **52.3** | **59.8** |
| | Std Dev | **1.1** | **3.2** | **5.0** | **10.0** |

| | | | | | |
|---|---|---|---|---|---|
| RpM IFN | Peg 400 d/a | 2.4 | 30.3 | 48.9 | 63.4 |
| | | 2.1 | 25.6 | 39.8 | 52.2 |
| | | 2.5 | 28.4 | 36.7 | 43.5 |
| | | 2.9 | 26.2 | 34.6 | 41.1 |
| | Mean | **2.5** | **27.6** | **40.0** | **50.1** |
| | Std Dev | **0.3** | **2.2** | **6.3** | **10.1** |

| | | | | | |
|---|---|---|---|---|---|
| **RpM IFN** | **Peg 4000-15d/a** | 1.4 | 32.9 | 54.8 | 62.8 |
| | | 2.1 | 32.7 | 50.2 | 57.8 |
| | Mean | **1.8** | **32.8** | **52.5** | **60.3** |
| | Std Dev | **0.5** | **0.1** | **3.3** | **3.5** |
| **RpM IFN** | **Peg 4000-7.5d/a** | 2.4 | 45 | 63.6 | 75.2 |
| | | 3.6 | 36.7 | 54.1 | 62.6 |
| | Mean | **3.0** | **40.9** | **58.9** | **68.9** |
| | Std Dev | **0.8** | **5.9** | **6.7** | **8.9** |
| **RpM IFN** | **Peg 8000-7.5d/a** | 1.4 | 28.4 | 44.2 | 58.8 |
| | | 1.6 | 21.7 | 37.6 | 49 |
| | Mean | **1.5** | **25.1** | **40.9** | **53.9** |
| | Std Dev | **0.1** | **4.7** | **4.7** | **6.9** |
| **RpH Ctrl** | **Peg 4000-15d/a** | | | | 3.3 |
| | | | | | 1.4 |
| | Mean | | | | **2.4** |
| | Std Dev | | | | **1.3** |
| **RpH Ctrl** | **Peg 2000 d/a** | | | | 1.6 |
| | | | | | 1.7 |
| | Mean | | | | **1.7** |
| | Std Dev | | | | **0.1** |
| **RpH IFN** | **Peg 4000-15d/a** | | | | 63.9 |
| | | | | | 62.3 |
| | Mean | | | | **63.1** |
| | Std Dev | | | | **1.1** |
| **RpH IFN** | **Peg 2000 d/a** | | | | 67.3 |
| | | | | | 67.9 |
| | Mean | | | | **67.6** |
| | Std Dev | | | | **0.4** |

**The numbers 1, 2, 3, & 4 indicate the hours after addition of the rickettsiae or mock-infection.**

| | | 1 | 2 | 3 | 4 |
|---|---|---|---|---|---|
| **Mk Ctrl** | **Peg 4000 a** | | 0.45 | 0.4 | 0.41 |
| | | | 0.45 | 0.4 | 0.3 |
| | Mean | | **0.5** | **0.4** | **0.4** |
| | Std Dev | | **0.0** | **0.0** | **0.1** |

| | | 1 | 2 | 3 | 4 |
|---|---|---|---|---|---|
| **RpL Ctrl** | **Peg 4000 a** | | 0.31 | 0.53 | 0.37 |
| | | | 0.81 | 0.51 | 0.36 |
| | Mean | | **0.6** | **0.5** | **0.4** |
| | Std Dev | | **0.4** | **0.0** | **0.0** |

| | | | | |
|---|---|---|---|---|
| **RpM Ctrl** | **Peg 4000 a** | 0.39 | 0.4 | 0.2 |
| | | 0.38 | 0.1 | 0.43 |
| | Mean | **0.4** | **0.3** | **0.3** |
| | Std Dev | **0.0** | **0.2** | **0.2** |
| **RpH Ctrl** | **Peg 4000 a** | 0.18 | 0.69 | 1.71 |
| | | 0.18 | 0.59 | 0.9 |
| | Mean | **0.2** | **0.6** | **1.3** |
| | Std Dev | **0.0** | **0.1** | **0.6** |
| **Mk IFN** | **Peg 4000 a** | 0.37 | 0.62 | 0.69 |
| | | 0.31 | 0.54 | 0.58 |
| | Mean | **0.3** | **0.6** | **0.6** |
| | Std Dev | **0.0** | **0.1** | **0.1** |

| | | | | |
|---|---|---|---|---|
| **RpL IFN** | **Peg 4000 a** | 10.15 | 15.79 | 18.92 |
| | | 12.33 | 17.93 | 19.85 |
| | Mean | **11.2** | **16.9** | **19.4** |
| | Std Dev | **1.5** | **1.5** | **0.7** |
| **RpM IFN** | **Peg 4000 a** | 15.42 | 21.64 | 23.57 |
| | | 18.35 | 25.45 | 28.4 |
| | Mean | **16.9** | **23.5** | **26.0** |
| | Std Dev | **2.1** | **2.7** | **3.4** |
| **RpH IFN** | **Peg 4000 a** | 25.24 | 32.72 | 35.06 |
| | | 25.47 | 31.46 | 31.36 |
| | Mean | **25.4** | **32.1** | **33.2** |
| | Std Dev | **0.2** | **0.9** | **2.6** |

| | | |
|---|---|---|
| ***Mk Ctrl*** | ***Peg 8000 a*** | 1.9 |
| | | 1.6 |
| | | 1.9 |
| | | 1.4 |
| | Mean | **1.7** |
| | Std Dev | **0.2** |

| | | 1 | 2 | 3 | 4 |
|---|---|---|---|---|---|
| ***Mk Ctrl*** | ***Peg 8000 a15*** | | | 1.2 | 1.5 |
| | | | | 1.1 | 1.7 |
| | | | | | 1.4 |
| | | | | | 1.4 |
| | Mean | | | **1.2** | **1.5** |
| | Std Dev | | | **0.1** | **0.1** |

| | | | |
|---|---|---|---|
| ***RpM Ctrl*** | ***Peg 8000 a*** | | 1.4 |
| | | | 1.5 |
| | | | 1.7 |
| | Mean | | **1.5** |
| | Std Dev | | **0.2** |

| | | | | |
|---|---|---|---|---|
| ***RpM Ctrl*** | ***Peg 8000 a15*** | | 1.2 | 1.5 |
| | | | 1.3 | 1.6 |
| | | | | 1.3 |
| | Mean | | **1.3** | **1.5** |
| | Std Dev | | **0.1** | **0.2** |

| | | | | | | | | |
|---|---|---|---|---|---|---|---|---|
| *Mk IFN* | *Peg 8000 a* | | 1.8 | *Mk IFN* | *Peg 8000 a15* | | 1.5 | 1.6 |
| | | | 1.9 | | | | 1.6 | 1.3 |
| | | | 1.5 | | | | | |
| | | | 1.2 | | | | | |
| | Mean | | **1.6** | | Mean | | **1.6** | **1.5** |
| | Std Dev | | **0.3** | | Std Dev | | **0.1** | **0.2** |

| | | | |
|---|---|---|---|
| *RpM IFN* | *Peg 8000 a* | | 2.9 |
| | | | 3.2 |
| | | | 2.8 |
| | Mean | | **3.0** |
| | Std Dev | | **0.2** |

| | | | | |
|---|---|---|---|---|
| *RpM IFN* | *Peg 8000 a15* | | 4.2 | 3.0 |
| | | | 5.3 | 2.8 |
| | | | | 2.6 |
| | Mean | | **4.8** | **2.8** |
| | Std Dev | | **0.8** | **0.2** |

**DEFINITION OF ABBREVIATIONS IN THIS TABLE**

**The numbers 1, 2, 3, & 4 indicate the hours after addition of the rickettsiae or mock-infection.**

**Mk Ctrl=Untreated, mock-infected**
**RpL Ctrl=Untreated, RpLow infected**
**RpM Ctrl=Untreated, RpMedium infected**
**RpH Ctrl=Untreated, RpHigh infected**

**Mk IFN=IFN-treated, mock-infected**
**RpL IFN=IFN-treated, RpLow infected**
**RpM IFN=IFN-treated, RpMedium infected**
**RpH IFN=IFN-treated, RpHigh infected**

**See Figure 3 and the results section for additional information about RpL, RpM, and RpH.**
**These indicate the use of different concentrations of rickettsiae.**

**"d/a" indicates that PEG was added at the time of infection or mock-infection**
**"a15" indicates that PEG was added 15 minutes after infection or mock-infection**
**"a" indicates that PEG was added 1 hour after infection or mock-infection**

**Pegs were generally used at 30 mM, except for Peg 8000, which was used at 15 mM.**
**In some instances, Peg 4000 was used at 15 mM & 7 mM; and Peg 8000 was used at 7.5 mM.**

**Peg 4000-15d/a indicates that Peg 4000 was used at 15 mM**
**Peg 4000-7.5d/a indicates that Peg 4000 was used at 7.5 mM**

**Peg 8000-7.5d/a indicates that Peg 8000 was used at 7.5 mM**

**TABLE S4, SHEET 2 (pages 15-18) -- PERCENTAGE OF TOTAL LDH RELEASED (means & standard deviations)**

**The next 4 pages represent Sheet 2 of Table S4. They provide a summary of some of the data (means and standard deviations) that are shown in Sheet 1 of Table S4.**

**The means below are highlighted in yellow.**

**The standard deviations below are highlighted in orange.**

| | | Hours 1 | 2 | 3 | 4 |
|---|---|---|---|---|---|
| Mk Ctrl | No addition | 0.2 | 0.4 | 0.4 | 0.7 |
| | | 0.2 | 0.4 | 0.6 | 0.5 |
| Mk Ctrl | Peg 1450 d/a | 0.0 | 0.0 | 0.0 | 0.2 |
| | | 0.0 | 0.0 | 0.0 | 0.2 |
| Mk Ctrl | Peg 2000 d/a | 0.0 | 0.0 | 0.0 | 0.4 |
| | | 0.0 | 0.0 | 0.0 | 0.4 |
| Mk Ctrl | Peg 4000 d/a | 1.3 | 1.7 | 1.5 | 1.2 |
| | | 1.5 | 0.9 | 1.0 | 1.0 |
| Mk Ctrl | Peg 8000 d/a | 2.2 | 1.7 | 1.8 | 2.1 |
| | | 0.6 | 0.4 | 0.5 | 0.7 |
| Mk Ctrl | Peg 4000 a | | 0.5 | 0.4 | 0.4 |
| | | | 0.0 | 0.0 | 0.1 |
| Mk Ctrl | Peg 8000 a | | | | 1.7 |
| | | | | | 0.2 |
| Mk Ctrl | Peg 8000 a15 | | | 1.2 | 1.5 |
| | | | | 0.1 | 0.1 |

| | | Hours 1 | 2 | 3 | 4 |
|---|---|---|---|---|---|
| RpM Ctrl | No addition | 0.3 | 0.6 | 0.6 | 0.8 |
| | | 0.4 | 0.4 | 0.7 | 0.4 |
| RpM Ctrl | Peg 400 d/a | 0.0 | 0.0 | 0.0 | 0.0 |
| | | 0.0 | 0.0 | 0.0 | 0.0 |
| RpM Ctrl | Peg 1450 d/a | 0.0 | 0.0 | 0.0 | 0.0 |
| | | 0.0 | 0.0 | 0.0 | 0.0 |
| RpM Ctrl | Peg 2000 d/a | 0.0 | 0.3 | 0.3 | 0.0 |
| | | 0.0 | 0.4 | 0.4 | 0.0 |
| RpM Ctrl | Peg 4000 d/a | 0.8 | 1.2 | 1.6 | 1.6 |
| | | 1.0 | 1.1 | 1.2 | 1.1 |
| RpM Ctrl | Peg 8000 d/a | 1.7 | 2.8 | 1.7 | 2.6 |
| | | 0.2 | 0.4 | 0.5 | 1.0 |
| RpM Ctrl | Peg 4000 a | | 0.4 | 0.3 | 0.3 |
| | | | 0.0 | 0.2 | 0.2 |
| RpM Ctrl | Peg 8000 a | | | | 1.5 |
| | | | | | 0.2 |
| RpM Ctrl | Peg 8000 a15 | | | 1.3 | 1.5 |
| | | | | 0.1 | 0.2 |

**Data for Figure 4**

**Percentage of Total LDH Released in IFN-treated C166 Cultures Infected with *R. prowazekii***

| Hours | No addition | | Peg 1450 d/a | | Peg 2000 d/a | | Peg 4000 d/a | | Peg 8000 d/a | |
|---|---|---|---|---|---|---|---|---|---|---|
| | Mean | Std dev | Mean | Std dev | Mean | Std dev | Mean | Std dev | Mean | Std dev |
| 1 | 3.9 | 2.9 | 2.7 | 1.1 | 2.9 | 2.0 | 1.0 | 1.2 | 2.3 | 0.4 |
| 2 | 41.9 | 3.8 | 37.2 | 3.2 | 44.5 | 0.9 | 18.1 | 2.4 | 3.2 | 0.8 |
| 3 | 55.2 | 5.2 | 52.3 | 5.0 | 59.1 | 8.0 | 30.9 | 2.1 | 4.6 | 1.0 |
| 4 | 58.1 | 8.8 | 59.8 | 10.0 | 65.7 | 14.1 | 35.9 | 4.7 | 5.0 | 1.7 |

| | | Hours 1 | 2 | 3 | 4 |
|---|---|---|---|---|---|
| Mk IFN | No addition | 0.4 | 0.4 | 0.6 | 0.7 |
| | | 0.5 | 0.7 | 0.6 | 0.6 |
| Mk IFN | Peg 1450 d/a | 0.0 | 0.0 | 0.0 | 0.0 |
| | | 0.0 | 0.0 | 0.0 | 0.0 |
| Mk IFN | Peg 2000 d/a | 0.0 | 0.0 | 0.0 | 0.5 |
| | | 0.0 | 0.0 | 0.0 | 0.7 |
| Mk IFN | Peg 4000 d/a | 1.2 | 2.1 | 4.1 | 4.8 |
| | | 1.6 | 1.7 | 1.1 | 2.5 |
| Mk IFN | Peg 8000 d/a | 2.3 | 2.3 | 2.0 | 2.7 |
| | | 0.4 | 0.3 | 0.5 | 1.2 |
| Mk IFN | Peg 4000 a | | 0.3 | 0.6 | 0.6 |
| | | | 0.0 | 0.1 | 0.1 |
| Mk IFN | Peg 8000 a | | | | 1.6 |
| | | | | | 0.3 |
| Mk IFN | Peg 8000 a15 | | | 1.6 | 1.5 |
| | | | | 0.1 | 0.2 |

| | | Hours 1 | 2 | 3 | 4 |
|---|---|---|---|---|---|
| RpM IFN | No addition | 3.9 | 41.9 | 55.2 | 58.1 |
| | | 2.9 | 3.8 | 5.2 | 8.8 |
| RpM IFN | Peg 400 d/a | 2.5 | 27.6 | 40.0 | 50.1 |
| | | 0.3 | 2.2 | 6.3 | 10.1 |
| RpM IFN | Peg 1450 d/a | 2.7 | 37.2 | 52.3 | 59.8 |
| | | 1.1 | 3.2 | 5.0 | 10.0 |
| RpM IFN | Peg 2000 d/a | 2.9 | 44.5 | 59.1 | 65.7 |
| | | 2.0 | 0.9 | 8.0 | 14.1 |
| RpM IFN | Peg 4000 d/a | 1.0 | 18.1 | 30.9 | 35.9 |
| | | 1.2 | 2.4 | 2.1 | 4.7 |
| RpM IFN | Peg 8000 d/a | 2.3 | 3.2 | 4.6 | 5.0 |
| | | 0.4 | 0.8 | 1.0 | 1.7 |
| RpM IFN | Peg 4000 a | | 16.9 | 23.5 | 26.0 |
| | | | 2.1 | 2.7 | 3.4 |
| RpM IFN | Peg 8000 a | | | | 3.0 |
| | | | | | 0.2 |
| RpM IFN | Peg 8000 a15 | | | 4.8 | 2.8 |
| | | | | 0.8 | 0.2 |

| | | Hours 1 | 2 | 3 | 4 |
|---|---|---|---|---|---|
| RpM IFN | No addition | 3.9 | 41.9 | 55.2 | 58.1 |
| | | 2.9 | 3.8 | 5.2 | 8.8 |
| RpM IFN | Peg 1450 d/a | 2.7 | 37.2 | 52.3 | 59.8 |
| | | 1.1 | 3.2 | 5.0 | 10.0 |
| RpM IFN | Peg 2000 d/a | 2.9 | 44.5 | 59.1 | 65.7 |
| | | 2.0 | 0.9 | 8.0 | 14.1 |
| RpM IFN | Peg 4000 d/a | 1.0 | 18.1 | 30.9 | 35.9 |
| | | 1.2 | 2.4 | 2.1 | 4.7 |
| RpM IFN | Peg 8000 d/a | 2.3 | 3.2 | 4.6 | 5.0 |
| | | 0.4 | 0.8 | 1.0 | 1.7 |
| RpM IFN | Peg 4000 a | | 16.9 | 23.5 | 26.0 |
| | | | 2.1 | 2.7 | 3.4 |
| RpM IFN | Peg 8000a | | | | 3.0 |
| | | | | | 0.2 |
| RpM IFN | Peg 8000a15 | | | 4.8 | 2.8 |
| | | | | 0.8 | 0.2 |
