# Supplementary material for: Involvement of Pore Formation and Osmotic Lysis in the Rapid Killing of Gamma Interferon-Pretreated C166 Endothelial Cells by Rickettsia prowazekii
Source: Trop Med Infect Dis. 2022 Aug 1;7(8):163. doi: 10.3390/tropicalmed7080163 (PMC9415803; doi:10.3390/tropicalmed7080163)
Supplement: Supplementary file 1 [file tropicalmed-07-00163-s001.zip › tropicalmed-1822686-supplementary/Table S5 Rickettsial Infection Data for Figure 3.pdf]

Table S5
Rickettsial Infection Data for Figure 3

	Untreated cells				IFN-treated cells			
	**%R**	**RI**	**NR*100**	**NR**	**%R**	**RI**	**NR*100**	**NR**
**RpLow**	63	3.1	195	2.0	73	5.4	393	3.9
	55	3.6	200	2.0	81	5.1	410	4.1
	74	4.5	332	3.3	80	7.5	596	6.0
**Avg**	64.0	3.7	242.3	2.4	78.0	6.0	466.3	4.7
**Std Dev**	9.5	0.7	77.7	0.8	4.4	1.3	112.6	1.1
**RpMedium**	82	5.9	482	4.8	91	8.0	729	7.3
	91	7.1	646	6.5	92	7.4	684	6.8
	80	7.6	609	6.1	97	12.1	1172	11.7
**Avg**	84.3	6.9	579.0	5.8	93.3	9.2	861.7	8.6
**Std Dev**	5.9	0.9	86.0	0.9	3.2	2.5	269.7	2.7
**RpHigh**	98	13.5	1323	13.2	95	13.3	1265	12.7
	88	10.0	880	8.8	97	16.0	1548	15.5
	97	17.5	1694	16.9	96	16.0	1532	15.3
**Avg**	94.3	13.7	1299.0	13.0	96.0	15.1	1448.3	14.5
**Std Dev**	5.5	3.7	407.5	4.1	1.0	1.5	159.0	1.6

%R= percent infected
RI=rickettsiae per infected cell
NR*100=rickettsiae in 100 cells
NR=rickettsiae per cell
