# Supplementary material for: Involvement of Pore Formation and Osmotic Lysis in the Rapid Killing of Gamma Interferon-Pretreated C166 Endothelial Cells by Rickettsia prowazekii
Source: Trop Med Infect Dis. 2022 Aug 1;7(8):163. doi: 10.3390/tropicalmed7080163 (PMC9415803; doi:10.3390/tropicalmed7080163)
Supplement: Supplementary file 1 [file tropicalmed-07-00163-s001.zip › tropicalmed-1822686-supplementary/Table S6 LDH Release with Lower PEG Concentrations.pdf]

Table S6
Percentage of Total LDH Released with Lower PEG Concentrations

Abbreviationa are defined on page 2

		Hours after addition of rickettsiae 1	2	3	4
RpM IFN	Peg 4000-30 mM d/a	0.04	18.57	29.4	32.04
		0	18.89	29.89	34.25
		1	15.7	34.2	40.6
		0.4	14.7	32.7	43.1
		3.1	20.7	30.3	33.4
		1.5	20.1	28.9	32.2
	Mean	**1.0**	**18.1**	**30.9**	**35.9**
	Std Dev	**1.2**	**2.4**	**2.1**	**4.7**
RpM IFN	Peg 4000-15 mM d/a	1.4	32.9	54.8	62.8
		2.1	32.7	50.2	57.8
	Mean	**1.8**	**32.8**	**52.5**	**60.3**
	Std Dev	**0.5**	**0.1**	**3.3**	**3.5**
RpM IFN	Peg 4000-7.5 mM d/a	2.4	45	63.6	75.2
		3.6	36.7	54.1	62.6
	Mean	**3.0**	**40.9**	**58.9**	**68.9**
	Std Dev	**0.8**	**5.9**	**6.7**	**8.9**
RpM IFN	No PEG	3.76	41.75	52.12	55.62
		2.81	43.3	54.58	58.46
		5	43.3	57.1	65.4
		3.9	47.5	63	74.3
		8.9	38.6	47.5	49.1
		9.5	36.9	43.8	46.1
		2.26		60.3	66.1
		3.02		58	61.4
		1.3		61.5	67.5
		1.2		58.6	62.1
		1.3		57.2	50
				59.9	49.4
				50.8	50
				53.1	
				53.2	
				52.4	
	Mean	**3.91**	**41.89**	**55.19**	**58.11**
	Std Dev	**2.88**	**3.77**	**5.23**	**8.82**

		Hours after addition of rickettsiae			
		1	2	3	4
RpM IFN	Peg 8000-15 mM d/a	2.8	4.1	3.3	4.8
		2.2	3.6	3.2	3.9
		2	2.5	3	3.5
		2	2.5	3.3	4.3
				3.9	4.7
				5.1	5.7
				5.5	5.1
				3.9	5.2
				4.6	8.5
				4.7	7.3
				5.2	5.7
				5	7.3
				5.1	3.4
				5.8	2.9
				6.5	3
				4.9	
	Mean	**2.3**	**3.2**	**4.6**	**5.0**
	Std Dev	**0.4**	**0.8**	**1.0**	**1.7**
RpM IFN	Peg 8000-7.5 mM d/a	1.4	28.4	44.2	58.8
		1.6	21.7	37.6	49
	Mean	**1.5**	**25.1**	**40.9**	**53.9**
	Std Dev	**0.1**	**4.7**	**4.7**	**6.9**

DEFINITION OF ABBREVIATIONS IN THIS TABLE

"d/a" indicates that PEGs were added along with the rickettsiae.

RpM IFN=IFN-treated, RpMedium infected

See Figure 3 and the results section for additional information about RpL, RpM, and RpH. These indicate the use of different concentrations of rickettsiae.
