# Supplementary material for: Involvement of Pore Formation and Osmotic Lysis in the Rapid Killing of Gamma Interferon-Pretreated C166 Endothelial Cells by Rickettsia prowazekii
Source: Trop Med Infect Dis. 2022 Aug 1;7(8):163. doi: 10.3390/tropicalmed7080163 (PMC9415803; doi:10.3390/tropicalmed7080163)
Supplement: Supplementary file 1 [file tropicalmed-07-00163-s001.zip › tropicalmed-1822686-supplementary/Table S7 LDH Release after removal of PEG.pdf]

Table S7<br><br>LDH release after removal of PEG	Abbreviations for treatments are shown in red.<br>Definitions are given in black, bold type<br>below the treatment abbreviations.<br><br>"d/a" indicates that Peg was added at the time of<br>infection or mock-infection

Expts 74 & 75
Exp 75

Treatment for 3-4 h after mock-infection or infection	Treatment post wash for 3-3.5 additional h	% total LDH released	Average	Std Dev	n
C+M<br>**Untreated<br>mock-infected**	FMS<br>**Phenol red free<br>medium+5% serum**	0.00<br>0.20<br>0.40	0.2	0.2	3
C+M+P8kDA<br>**Untreated<br>mock-infected<br>+Peg 8000 d/a**	FMS<br>**Phenol red free<br>medium+5% serum**	0.00<br>0.10<br>0.80<br>0.40	0.3	0.4	4
C+M+P8kDA<br>**Untreated<br>mock-infected<br>+Peg 8000 d/a**	P8k<br>**Peg 8000**	1.50<br>1.50	1.5	0.0	2
I+M<br>**IFN-treated<br>mock-infected**	FMS<br>**Phenol red free<br>medium+5% serum**	0.00<br>0.70<br>0.20<br>0.60	0.4	0.3	4
I+M+P8kDA<br>**IFN-treated<br>mock-infected<br>+Peg 8000 d/a**	FMS<br>**Phenol red free<br>medium+5% serum**	0.00<br>2.30<br>3.20	1.8	1.7	3
I+M+P8kDA<br>**IFN-treated<br>mock-infected<br>+Peg 8000 d/a**	P8k<br>**Peg 8000**	1.80<br>1.90	1.9	0.1	2
C+Rp<br>**Untreated<br>Infected**	FMS<br>**Phenol red free<br>medium+5% serum**	0.20<br>0.10<br>0.30<br>0.50	0.3	0.2	4
C+Rp+P8kDA<br>**Untreated<br>Infected<br>+Peg 8000 d/a**	FMS<br>**Phenol red free<br>medium+5% serum**	0.30<br>0.40<br>0.50<br>1.00<br>0.50<br>0.80	0.6	0.3	6
C+Rp+P8kDA<br>**Untreated<br>Infected<br>+Peg 8000 d/a**	P8k<br>**Peg 8000**	1.80<br>1.50	1.7	0.2	2
I+Rp<br>**IFN-treated<br>Infected**	FMS<br>**Phenol red free<br>medium+5% serum**	7.6<br>7.9<br>9.1<br>9.4	8.5	0.9	4
I+Rp+P8kDA<br>**IFN-treated<br>Infected<br>+Peg 8000 d/a**	FMS<br>**Phenol red free<br>medium+5% serum**	19.7<br>21.9<br>20.2<br>22.7<br>23.1<br>23.1	21.8	1.5	6
I+Rp+P8kDA<br>**IFN-treated<br>Infected<br>+Peg 8000 d/a**	P8k<br>**Peg 8000**	2.4<br>2.5	2.5	0.1	2
