# Supplementary figures and images for: Involvement of Pore Formation and Osmotic Lysis in the Rapid Killing of Gamma Interferon-Pretreated C166 Endothelial Cells by Rickettsia prowazekii
Source: Trop Med Infect Dis. 2022 Aug 1;7(8):163. doi: 10.3390/tropicalmed7080163 (PMC9415803; doi:10.3390/tropicalmed7080163)

Figure S1, Results of ethidium bromide staining in exp 77 (infected)

Exp 77 - Infected

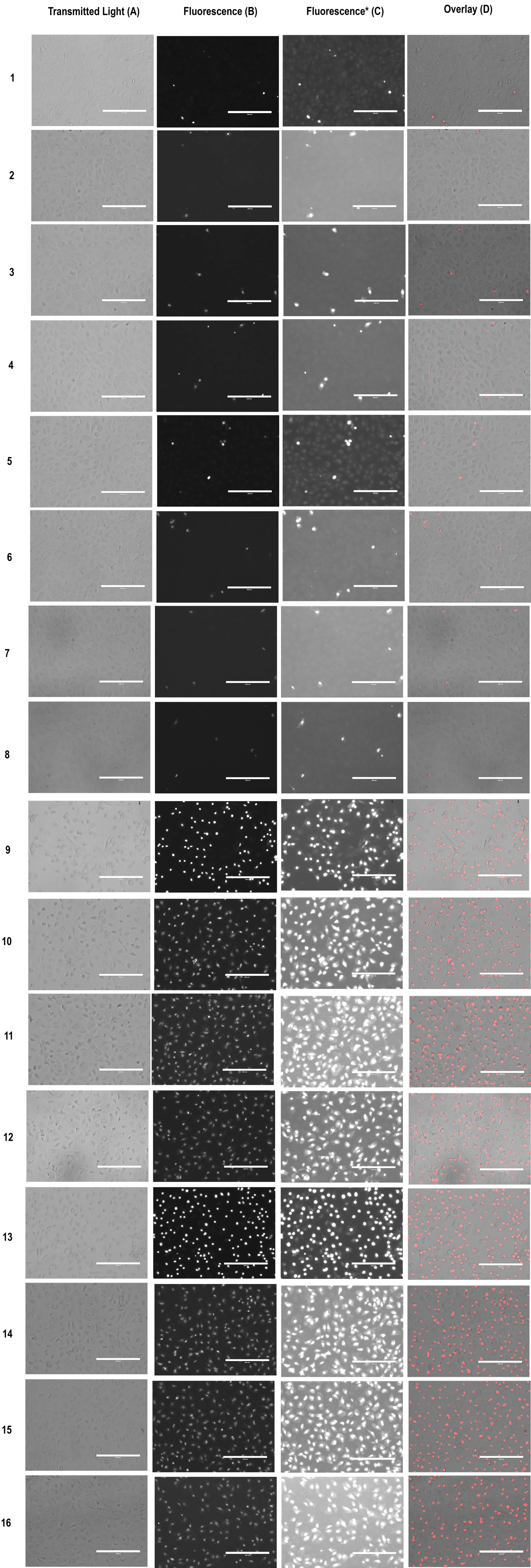

Supplement: Supplementary file 1 [file tropicalmed-07-00163-s001.zip › tropicalmed-1822686-supplementary/Figure S1, Ethidium bromide staining exp 77 (infected).pdf]
