# Supplementary material for: Involvement of Pore Formation and Osmotic Lysis in the Rapid Killing of Gamma Interferon-Pretreated C166 Endothelial Cells by Rickettsia prowazekii
Source: Trop Med Infect Dis. 2022 Aug 1;7(8):163. doi: 10.3390/tropicalmed7080163 (PMC9415803; doi:10.3390/tropicalmed7080163)

Figure S1, Results of ethidium bromide staining in experiment 77

Exp 77 - Mock-infected

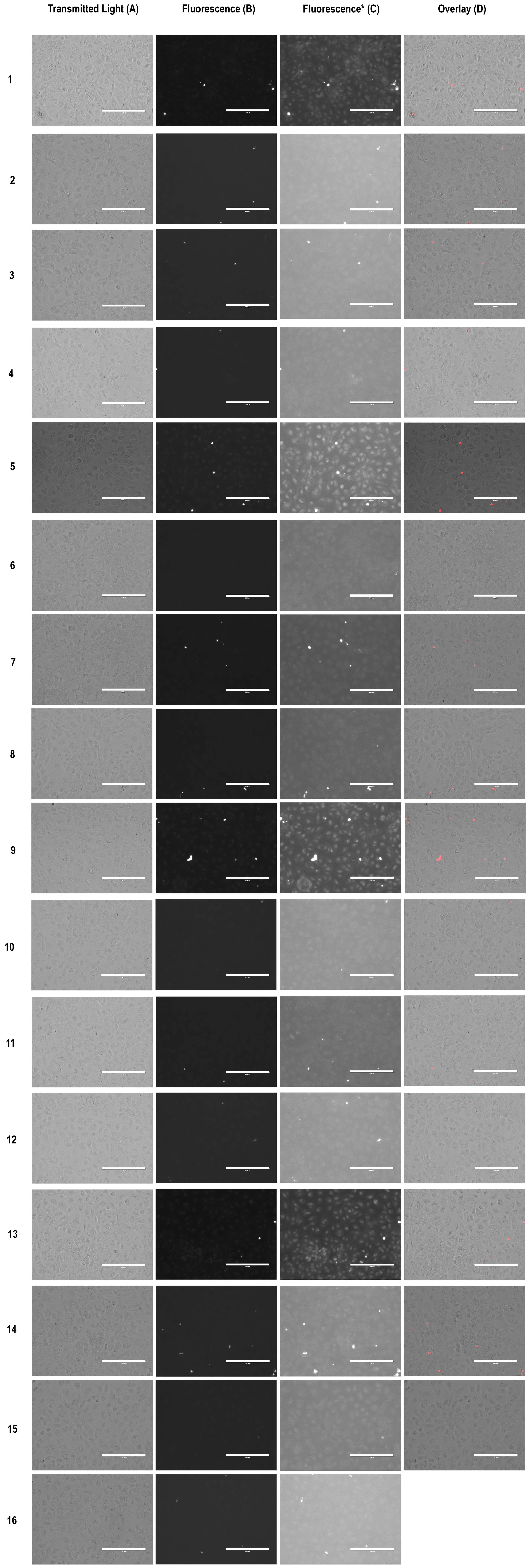

Supplement: Supplementary file 1 [file Figure S1, Ethidium bromide staining exp 77 (mock-infected).pdf › tropicalmed-1822686-supplementary/Figure S1, Ethidium bromide staining exp 77 (mock-infected).pdf]
