# Supplementary material for: Involvement of Pore Formation and Osmotic Lysis in the Rapid Killing of Gamma Interferon-Pretreated C166 Endothelial Cells by Rickettsia prowazekii
Source: Trop Med Infect Dis. 2022 Aug 1;7(8):163. doi: 10.3390/tropicalmed7080163 (PMC9415803; doi:10.3390/tropicalmed7080163)

Figure S2, Results of ethidium bromide staining in exp 79

Exp 79 - Infected

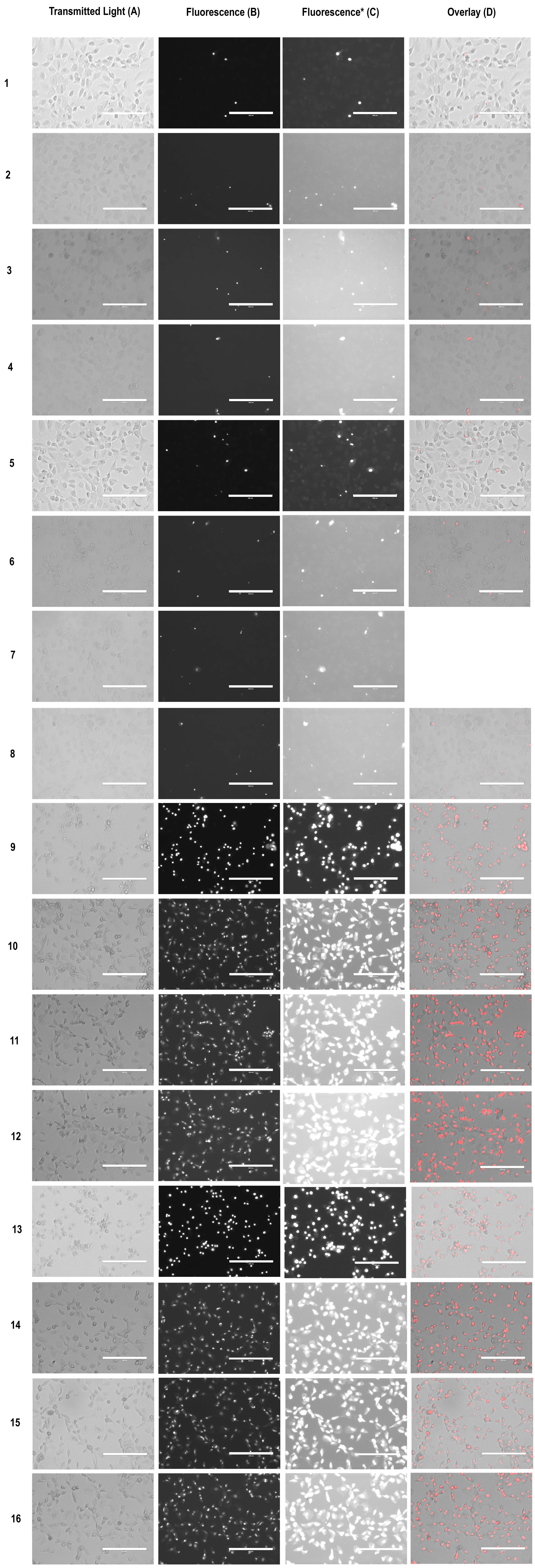

Supplement: Supplementary file 1 [file Figure S2, Ethidium bromide staining exp 79 (infected).pdf › tropicalmed-1822686-supplementary/Figure S2, Ethidium bromide staining exp 79 (infected).pdf]
